# Meta-analysis of viscosity of aqueous deep eutectic solvents and their components

**DOI:** 10.1038/s41598-020-78101-y

**Published:** 2020-12-07

**Authors:** Gudrun Gygli, Xinmeng Xu, Jürgen Pleiss

**Affiliations:** 1 Institute for Biological Interfaces (IBG 1), Karlsruhe Institute of Technology (KIT), Hermann-von-Helmholtz-Platz 1, 76344 Karlsruhe, Germany; 2 Institute of Biochemistry and Technical Biochemistry, University of Stuttgart, Allmandring 31, 70569 Stuttgart, Germany

**Keywords:** Biotechnology, Chemistry

## Abstract

Deep eutectic solvents (DES) formed by quaternary ammonium salts and hydrogen bond donors are a promising green alternative to organic solvents. Their high viscosity at ambient temperatures can limit biocatalytic applications and therefore requires fine-tuning by adjusting water content and temperature. Here, we performed a meta-analysis of the impact of water content and temperature on the viscosities of four deep eutectic solvents (glyceline, reline, *N*,*N*-diethylethanol ammonium chloride-glycerol, *N*,*N*-diethylethanol ammonium chloride-ethylene glycol), their components (choline chloride, urea, glycerol, ethylene glycol), methanol, and pure water. We analyzed the viscosity data by an automated workflow, using Arrhenius and Vogel–Fulcher–Tammann–Hesse models. The consistency and completeness of experimental data and metadata was used as an essential criterion of data quality. We found that viscosities were reported for different temperature ranges, half the time without specifying a method of desiccation, and in almost half of the reports without specifying experimental errors. We found that the viscosity of the pure components varied widely, but that all aqueous mixtures (except for reline) have similar excess activation energy of viscous flow ${E}^{excess}_{\eta}$= 3–5 kJ/mol, whereas reline had a negative excess activation energy (${E}^{excess}_{\eta}$= − 19 kJ/mol). The data and workflows used are accessible at 10.15490/FAIRDOMHUB.1.STUDY.767.1.

## Introduction

Deep eutectic solvents (DES) are mixtures of a quaternary ammonium salt and a hydrogen bond donor, which are of growing interest in biocatalysis and chemistry as a green alternative to organic solvents^1–3^. Their melting points are lower than that of their components, and they readily mix with water. Because DESs are efficient solvents for hydrophobic substrates and benign towards enzymes^4–6^, they are promising media for enzyme catalyzed reactions under non-aqueous conditions. Unfortunately, their high viscosity at ambient temperatures limits their applicability in biocatalysis. Adding small amounts of water or increasing the temperature decreases the viscosity and increases catalytic activity^6–8^. However, catalytic activity has a sharp optimum of water content and temperature: at high water content, undesired side reactions involving water becomes limiting, and at high temperature catalytic activity decrease due to thermal inactivation of the enzyme^9^. Therefore, the dependency of viscosity on temperature and on water content is crucial for designing biocatalytic processes with DES. Another crucial parameter for designing DES is the molar ratio of the DES components. While the temperature dependency of viscosity can be modelled phenomenologically using the linear Arrhenius model or the Vogel–Fulcher–Tamman–Hesse model (VFT)^10–12^, no general model for the deviation of the viscosity of aqueous DES–water mixtures from ideal mixing is available.

Experimentally determined viscosities of DES–water mixtures under varying water content and temperature are becoming more prevalent^13–36^. Due to the wealth of data available, it is now possible to perform meta-analyses of different mixtures at different temperatures and water contents. Meta-analyses are common practice in the health and environmental sciences^37^. Meta-analyses profit from complete and accessible data, from data quality estimates, and from community standards for data reporting. Completeness and standardization are crucial for the reporting of metadata such as the experimental methods, information about the devices used for viscosity measurement, the temperature, the pressure, and the units of the reported values. Complete reporting of data and metadata is also essential for quality control^38^.

Low quality data and incomplete reporting of experimental methods are the two major reasons for the observed reproducibility crisis^39^. Community standards such as the STRENDA guidelines for reporting of enzyme-catalyzed reactions^40,41^ or the STROBE checklists for reporting of epidemiology data^42,43^ have been proposed, but still are not fully accepted by the scientific community. Enforcing guidelines upon publication was successful to improve quality and reproducibility of crystal structure data in the Protein Data Bank (PDB)^44,45^, but required cooperation between the scientific community and the scientific journals.

Meta-analyses would greatly benefit from machine readable data, thus automating the selection of relevant sources and the extraction of data and metadata from sources. Machine readable data can be collected and analyzed by automated workflows, therefore replacing time intensive and error prone manual search, extraction, and analysis of data. Consequently, machine readability and automation is crucial to guarantee completeness and consistency of data as proposed by the F.A.I.R. guidelines (Findable, Accessible, Interoperable, Reusable)^46^. Therefore, data should not be hidden in publications as plain text, tables, or figures. Instead, data and metadata should be reported in an exchange format such as XML, which allows data to be linked to dictionaries containing pre-defined ontologies. The Chemical Markup Language (CML) has been developed to represent chemical information^47^ and has been used previously to store structured data on the density, viscosity, conductivity, and water activity of DES^48^.

In this study, published data on the viscosity of aqueous solution of two salts (choline chloride, ChCl and *N*,*N*-diethylethanol ammonium chloride, DAC), three hydrogen bond donors (urea, glycerol, and ethylene glycol), and the respective DESs were collected and systematically analyzed. For comparison, pure water and aqueous methanol mixtures were included in the analysis. To our knowledge, this is the first time that viscosity data from a large number of aqueous DES mixtures at different temperatures have been collected, compared, and consistently analyzed by an Arrhenius model and the Vogel–Fulcher–Tamman–Hesse model, thus demonstrating the challenges of data quality and validation methods and the value of data integration and analysis^48^.

## Results

The viscosity data on water and aqueous mixtures of methanol, of five DESs, and of four DES components were retrieved from literature (Table 1). Data covers the whole range of water content from *χ*_*w*_ = 0.0 to *χ*_*w*_ = 1.0, except for aqueous mixtures of urea, ChCl, DEACG, and DEACEG, and a temperature range from 293.15 to 449.85 K (Supplementary Figure S1). However, not all data covers the complete range, except for the narrow temperature range from 308.15 to 318.15 K, for which viscosity data exists for all mixtures. All data, analysis results, and workflows applied for analysis and visualization are available at FAIRDOMHub (10.15490/FAIRDOMHUB.1.STUDY.767.1).

**Table 1:** Ranges of χ_w_ and temperature for viscosities of 10 aqueous mixtures as collected from literature.

Component or DES	*χ*_*w*_ range	Temperature range (K)	References
Ethylene glycol	0.00–1.00	283.15–449.85	^24–26^
Methanol	0.00–1.00	278.15–323.15	^30–32^
Glycerol	0.00–1.00	243.15–373.15	^27–29^
Urea	0.86–0.98	308.15–328.15	^34^
Choline chloride	0.78–1.00	278.15–318.15	^23^
Choline chloride:urea (reline)	0.00–1.00	283.15–363.15	^19–22,49^
*N*,*N*-diethylethanol ammonium chloride: ethylene glycol (DEACEG)	0.00–0.9	298.15–343.15	^33^
*N*,*N*-diethylethanol ammonium chloride:glycerol (DEACG)	0.00–0.9	298.15–343.15	^33^
Choline chloride:glycerol (glyceline)	0.00–1.00	278.15–363.15	^13–20^
Choline chloride:ethylene glycol (ethaline)	0.00	293.15	^13^

### Pure water and aqueous methanol mixtures

Viscosity data for pure water was collected for a temperature range from 243.15 to 449.85 K from the sources cited in Table 1 (viscosity at *χ*_*w*_ = 1.0) and two additional sources^35,36^. Over the complete temperature range, the VFT model represents the data better than the Arrhenius model due to the curvature of the ln*η* − 1/T curve (Supplementary Figure S2). The Arrhenius model results in ln*η*_*0*_ and *E*_*η*_ values of − 6.6 ± 0.2 and 16.2 ± 0.6 kJ/mol, respectively (Supplementary Figure S2A, Supplementary  File “Arrhenius_water.csv”), the VFT model in A = -3.3, B = 502.3, T0 = 154.9 (Supplementary Figure S2B, Supplementary File “VFT_water.csv”).

Viscosity data for aqueous methanol mixtures was available for different temperature ranges, and no source specified whether methanol was desiccated before mixing. Data from different sources collected under identical conditions for *χ*_*w*_ = 0.0 and *χ*_*w*_ = 1.0 was combined, resulting in a larger temperature range (Supplementary Figure S3A). All straight lines resulting from the Arrhenius model intersect, which is a criterion of data quality. Arrhenius fits were excellent, with R^2^ values of 0.99 (SI file “Arrhenius_methanol.csv” for all parameters of the Arrhenius fits). The slopes of the fits and the resulting values for *E*_*η*_ were sensitive to individual data points due to the small number of available data (Supplementary Figure S3A). ln*η*_*0*_ had a minimum at *χ*_*w*_ = 0.7–0.8 (Supplementary Figure S3D). *E*_*η*_ increased almost linearly from 10.3 kJ/mol for pure methanol to 20.0–21.4 kJ/mol at *χ*_*w*_ = 0.7–0.8 (Supplementary Figure S3E) and decreased to 16.9 kJ/mol at *χ*_*w*_ = 1.0 (pure water). The *E*_*η*_ values positively deviated from an ideal mixture. This positive deviation is also reflected in ${E}^{excess}_{\eta}$, which was fit by a 4th order polynomial (Supplementary Figure S3C). ln*η*_*0*_ and *E*_*η*_ are anticorrelated (Supplementary Figure S3F, Supplementary File “Correlation_Arrheniusparameters_methanol.csv”). Substantial deviations of the values of *E*_*η*_ derived from two sources^31,32^ were observed at *χ*_*w*_ = 0.7–0.9 (Supplementary Figure E). These deviations are due to the consistently steeper slopes obtained by the Arrhenius fits for the data from^31^ compared to the other data. Notably, the 4th order fit of ${E}^{excess}_{\eta}$ fits better to the data from^32^ (Supplementary Figure S3C). Fits using the VFT model resulted in excellent fits (R^2^ = 0.99), but not only with convex curvature, but also with almost linear and even concave curvature (Supplementary Figure S3B, see Supplementary File “VFT_methanol.csv” and Supplementary Figure S4A–C for all VFT-parameters (A, B and T_0_, respectively).

### Aqueous binary mixtures of DES components

Aqueous solutions of ChCl and urea are limited by the solubility of the salts in water, leading to a narrow range of χ_w_ that was studied (Table 1). Viscosity data for ethaline was only available for one temperature and the pure DES. Therefore, no further analysis was performed for these mixtures.

Viscosity data for aqueous glycerol mixtures was available for different temperature ranges and no source specified whether glycerol was desiccated before mixing (Supplementary Figures S5A, S6A, S7A). By combining data from different sources collected under identical *χ*_*w*_ in the range 0.5–1.0, a larger temperature range was covered (Supplementary Figure S5). Performing Arrhenius modelling for different temperature ranges resulted in different fits. For each range, the fits were excellent (SI file “Arrhenius_glycerol.csv” for all parameters of the Arrhenius fits). All straight lines resulting from the Arrhenius model intersected for data from the same source. The source of the data influenced the slopes of the fits, and therefore ln*η*_*0*_ and *E*_*η*_ values, ${E}^{excess}_{\eta}$ and the relationship between ln*η*_*0*_ and *E*_*η*_ (Supplementary Figure S5C–F, Supplementary File “Correlation_Arrheniusparameters_glycerol.csv”,). Therefore, a separate analysis was performed for data from each source (Supplementary Figures S6, S7). ln*η*_*0*_ and *E*_*η*_ values were calculated from the Arrhenius fits using Eq. (4) (Supplementary Figures S6D,E, S7D,E). *E*_*η*_ decreased with increasing χ_w_ with a slightly concave curvature (Supplementary Figures S6E, S7E). For data from Sheely et al. *E*_*η*_ was 63.9 kJ/mol for pure glycerol and 17.3 kJ/mol for pure water^28^ (Supplementary Figure S6E). For data from ﻿Segur et al., *E*_*η*_ was 56.3 kJ/mol for pure glycerol and 15.4 kJ/mol for pure water^27^ (Supplementary Figure S7E). The positive deviation of *E*_*η*_ from an ideal mixture was reflected in a positive ${E}^{excess}_{\eta}$, which was fit by a 4th order polynomial (Supplementary Figures S6C, S7C). ln*η*_*0*_ and *E*_*η*_ were anticorrelated (Supplementary Figures S6F, S7F, “Correlation_Arrheniusparameters_glycerol_DOI1.csv”, “Correlation_Arrheniusparameters_glycerol_DOI2.csv”). The two series of *E*_*η*_ values can be explained by the consistently steeper slopes of the Arrhenius fits for the data from Sheely et al.^28^ as compared to the data from Segur et al.^27^. The deviation from an ideal mixture (${E}^{excess}_{\eta}$) was smallest for data from Segur et al.^27^ (Supplementary Figure S7C, see Supplementary Figures S5C, S6C for comparison). The size of the error bars depended on the number of data points available to perform Arrhenius fits, resulting in larger error bars if more data is available (Supplementary Figures S6D,E vs S7D,E). The VFT model resulted in excellent fits (Supplementary Figures S5B, S6B, S7B, see Supplementary File “VFT_glycerol.csv”, “VFT_glycerol_DOI1.csv”, “VFT_glycerol_DOI2.csv” and Supplementary Figures S8A–C for all VFT-parameters (A, B and T_0_, respectively) of all data, S8D, E, and F for data from Sheely et al.^28^ and S8G, H and I for data from Segur et al.^27^).

Viscosity data for aqueous ethylene glycol mixtures was available for different temperature ranges, and data from Sun et al.^25^ covered the highest temperatures (Supplementary Figure S9A). Only one source^24^ specified how ethylene glycol was desiccated before mixing (Supplementary Figures S9A,B, S10A,B). Data from different sources collected under identical *χ*_*w*_ (0.9–1.0) was combined (Supplementary Figure S9A). Arrhenius fits were excellent (SI file “Arrhenius_ ethylene glycol.csv” for all parameters of the Arrhenius fits). Straight lines resulting from the Arrhenius model intersected for data from the same source.

The source of the data influenced the slopes of the fits, and therefore ln*η*_*0*_ and *E*_*η*_ values, ${E}^{excess}_{\eta}$ and the relationship between ln*η*_*0*_ and *E*_*η*_ (Supplementary Figure S9C–F, Supplementary File “Correlation_Arrheniusparameters_ethylene glycol.csv”). Therefore a separate analysis was performed for data from Yang et al.^24^ (Supplementary Figure S10). ln*η*_*0*_ and *E*_*η*_ values were calculated from the Arrhenius fits using Eq. (4) (Supplementary Figure S10D,E). *E*_*η*_ was 27.4 kJ/mol for pure ethylene glycol and 14.8 kJ/mol for pure water (Supplementary Figure S10E). The *E*_*η*_ values deviated from an ideal mixture (Supplementary Figure S10E). The positive deviation was reflected in ${E}^{excess}_{\eta}$, which was fit by a 4th order polynomial (Supplementary Figure S10C). ln*η*_*0*_ and *E*_*η*_ were anticorrelated (Supplementary Figure S10F Supplementary File “Correlation_Arrheniusparameters_ethylene glycol_DOI1.csv”). The VFT model resulted in excellent fits (Supplementary Figure S10B, see Supplementary File “VFT_ethylene glycol_DOI1.csv” and Supplementary Figure S11A–C for all VFT-parameters (A, B and T_0_, respectively) of all data, S11D, E, and F for data from Yang et al.^24^).

### DES mixtures

**Figure 1:**
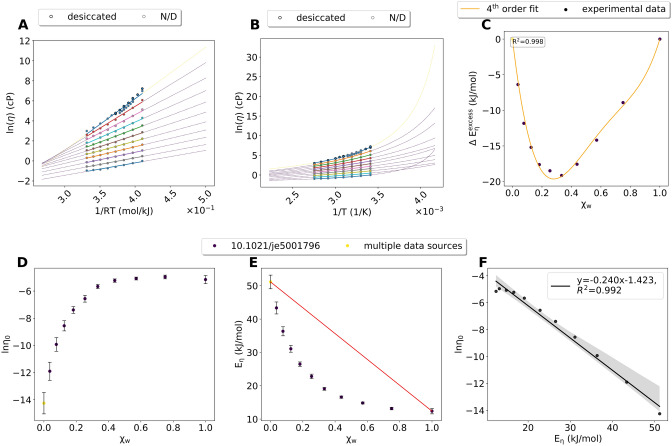
Reline–water mixtures. (**A**) Arrhenius fits (using a minimum of 3 data points). Dots and thick lines are the experimental data and the respective fit. The dashed lines are extensions of the fit. Colors of the dashed lines indicate the source of the data. Yellow: multiple data points from different sources were be combined. (**B**) VFT fits using a minimum of 4 data points. (**C**) ${E}^{excess}_{\eta}$ calculated based on the red line in (**E**). Colors of the data points indicate the source of the data. (**D**) ln*η*_*0*_ at different *χ*_*w*_. Error bars are calculated based on the fit in (A). Colors of the dashed lines indicate source of the data. (**E**) *E*_*η*_ at different *χ*_*w*_. The red line indicates the behavior of an ideal binary mixture and was used to calculate ${E}^{excess}_{\eta}$. (**F**) Correlation between ln*η*_*0*_ and *E*_*η*_.

Viscosity data for aqueous reline mixtures was available mostly from one source^21^, but multiple sources reported data for pure reline. Only one source^49^ specified how the DES components were desiccated before mixing (Fig. 1A,B). Arrhenius fits were excellent (R^2^ = 0.99, Supplementary File “Arrhenius_ reline.csv” for all parameters of the Arrhenius fits), and all straight lines resulting from the Arrhenius model intersected (Fig. 1A). ln*η*_*0*_ and *E*_*η*_ values were calculated from the Arrhenius fits using Eq. (4) (Fig. 1D,E). *E*_*η*_ was 51.2 kJ/mol for pure reline and 12.4 kJ/mol for pure water, and the values deviated considerably from an ideal mixture (Fig. 1E). The *E*_*η*_ deviations resulted in negative values for ${E}^{excess}_{\eta}$, which was fit by a 4th order polynomial (Fig. 1C). ln*η*_*0*_ and *E*_*η*_ were anticorrelated (Fig. 1F, Supplementary File “Correlation_Arrheniusparameters_reline.csv”). Fits using the VFT model were excellent (R^2^ = 0.99, Fig. 1B, see Supplementary File “VFT_reline.csv” and Supplementary Figures S12A, B, and C for all VFT-parameters (A, B and T_0_, respectively).

**Figure 2:**
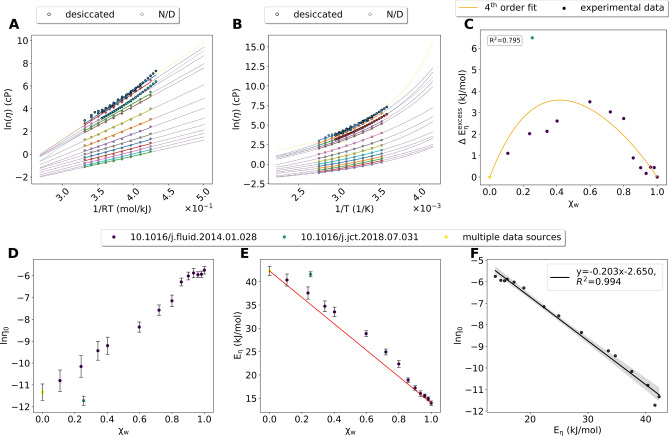
Glyceline–water mixtures. (**A**) Arrhenius fits (using a minimum of 3 data points). Dots and thick lines are the experimental data and the respective fit. The dashed lines are extensions of the fit. Colors of the dashed lines indicate the source of the data. Yellow: multiple data points from different sources were be combined. (**B**) VFT fits using a minimum of 4 data points. (**C**) ${E}^{excess}_{\eta}$, calculated based on the red line in (**E**). Colors of the data points indicate the source of the data. (**D**) ln*η*_*0*_ at different *χ*_*w*_. Error bars are calculated based on the fit in (**A**). Colors of the dashed lines indicate source of the data. (**E**) E_*η*_ at different *χ*_*w*_, same logic as (**D**). The red line indicates the behavior of an ideal binary mixture and was used to calculate ${E}^{excess}_{\eta}$. (**F**) Correlation between ln*η*_*0*_ and *E*_*η*_.

Viscosity data for glyceline–water mixtures was available mostly from one source^15^, but multiple sources reported data for pure glyceline. Only one source^16^ specified how the DES components were desiccated before mixing (Fig. 2A,B). Arrhenius fits were excellent (SI file “Arrhenius_glyceline.csv” for all parameters of the Arrhenius fits), and all straight lines resulting from the Arrhenius model intersected. ln*η*_*0*_ and *E*_*η*_ values were calculated from the Arrhenius fits using Eq. (4) (Fig. 2D,E). *E*_*η*_ was 42.3 kJ/mol for pure glyceline and 14.0 kJ/mol for pure water (Fig. 2E). The *E*_*η*_ values deviated from an ideal mixture (Fig. 2E), resulting in positive values of ${E}^{excess}_{\eta}$ (Fig. 2C). The data could not be fitted by a 4th order polynomial fit of good quality, mainly due to an outlier from one source^16^ (Fig. 2C). ln*η*_*0*_ and *E*_*η*_ were anticorrelated (Fig. 2F, Supplementary File “Correlation_Arrheniusparameters_glyceline.csv”). Fits using the VFT model were excellent (Fig. 2B, see Supplementary File “VFT_glyceline.csv” and Supplementary Figure S13A–C for all VFT-parameters (A, B and T_0_, respectively).

Viscosity data for aqueous DEACG mixtures was available from a single source^33^ (Supplementary Figure S14A). Arrhenius fits were excellent (SI file “Arrhenius_DEACG.csv” for all parameters of the Arrhenius fits), and all straight lines resulting from the Arrhenius model intersected. ln*η*_*0*_ and *E*_*η*_ values were calculated from the Arrhenius fits using Eq. (4) (Supplementary Figure S14D,E). *E*_*η*_ was 46.7 kJ/mol for pure DEACG and 19.1 kJ/mol for *χ*_*w*_ = 0.9 (Supplementary Figure S14E). The *E*_*η*_ values deviated from an ideal mixture (Supplementary Figure S14E), resulting in positive values of ${E}^{excess}_{\eta}$, which were fit by a 4th order polynomial (Supplementary Figure S14C). ln*η*_*0*_ and *E*_*η*_ were anticorrelated (Supplementary Figure S14F, Supplementary File “Correlation_Arrheniusparameters_DEACG.csv”). Fits using the VFT model were excellent [Supplementary Figure S14B, see Supplementary File “VFT_DEACG.csv” and Supplementary Figure S15A–C for all VFT-parameters (A, B and T_0_, respectively)].

Viscosity data for aqueous DEACEG mixtures was available from a single source^33^ (Supplementary Figure S16A). Arrhenius fits were excellent (SI file “Arrhenius_DEACEG.csv” for all parameters of the Arrhenius fits), and all straight lines resulting from the Arrhenius model intersected. ln*η*_*0*_ and *E*_*η*_ were calculated from the Arrhenius fits using Eq. (4) (Supplementary Figure S16D,E). *E*_*η*_ was 30.4 kJ/mol for pure DEACEG and 17.3 kJ/mol for *χ*_*w*_ = 0.9 (Supplementary Figure S16E). The *E*_*η*_ values deviated from an ideal mixture (Supplementary Figure S16E), resulting in positive values of ${E}^{excess}_{\eta}$, which were fit by a 4th order polynomial (Supplementary Figure S16C). ln*η*_*0*_ and *E*_*η*_ were anticorrelated (Supplementary Figure S16F, Supplementary File “Correlation_Arrheniusparameters_DEACEG.csv”), but the quality of the fit was influenced by deviating data points at low and high *E*_*η*_. The 4th order fit of ${E}^{excess}_{\eta}$ was excellent (Supplementary Figure S16C). Fits using the VFT model were excellent [Supplementary Figure S16B, see Supplementary File “VFT_DEACEG.csv” and Supplementary Figure S17A–C for all VFT-parameters (A, B and T_0_, respectively)].

## Discussion

Experimental data on viscosity of aqueous DES mixtures and their components was found for the whole range of *χ*_*w*_ between 0 and 1 (except for urea, ChCl, DEACG, and DEACEG), though the temperature ranges of each source differed and overlapped only for a narrow region between 308.15 and 318.15 K. Because ln*η* was not strictly linear in *T*^−1^, but slightly convex, *E*_*η*_ and ln*η*_*0*_ as obtained by the Arrhenius model depended on the analyzed temperature range. Therefore, for methanol, glycerol, and ethylene glycol mixtures, separate data analyses were performed for datasets from different sources, resulting in different dependencies of *E*_*η*_*(χ*_*w*_*)* and ln*η*_*0*_*(χ*_*w*_*).*

Fitting ln*η−*1/T data by an Arrhenius model requires viscosity to be measured for at least three different temperatures. However, combining data from different sources to derive *E*_*η*_(*χ*_*w*_) and ln*η*_*0*_(*χ*_*w*_) was not always possible, because the values of *χ*_*w*_ at which viscosity was measured differed between the sources by more than 0.05. As a consequence, for many mixtures the number of different temperatures reported was too small for a reliable analysis, resulting in a considerable loss of data during analysis. For aqueous glyceline mixtures, data was collected from eight different sources (SI, exp_ChCl_glycerol.csv), but only data from two sources could be used for the analyses by the Arrhenius model. Therefore, guidelines for a more systematic exploration of temperature ranges and a minimal number of data points to report are needed for compatibility between data from different sources, which then can be used for a consistent data analysis.

A major experimental challenge is the high hygroscopy of DES and the sensitive dependence of viscosity on the water content, especially at *χ*_*w*_ close to 0^50–52^. However, only half of the sources reported the method of desiccation of the DES components prior to experimentation. For glyceline–water mixtures, data from sources which reported the desiccation method and from sources which did not report the method were consistent (Fig. 2), whereas for aqueous glycerol and ethylene glycol mixtures, the lack of reporting the desiccation method resulted in outliers (Supplementary Figures S5 or S9) or substantial deviations in data from different sources (e.g. methanol–water mixtures, Supplementary Figure S3). Therefore, we support previous calls for community standards on measurement protocols and the complete reporting of metadata to ensure reproducibility^39^.

**Figure 3:**
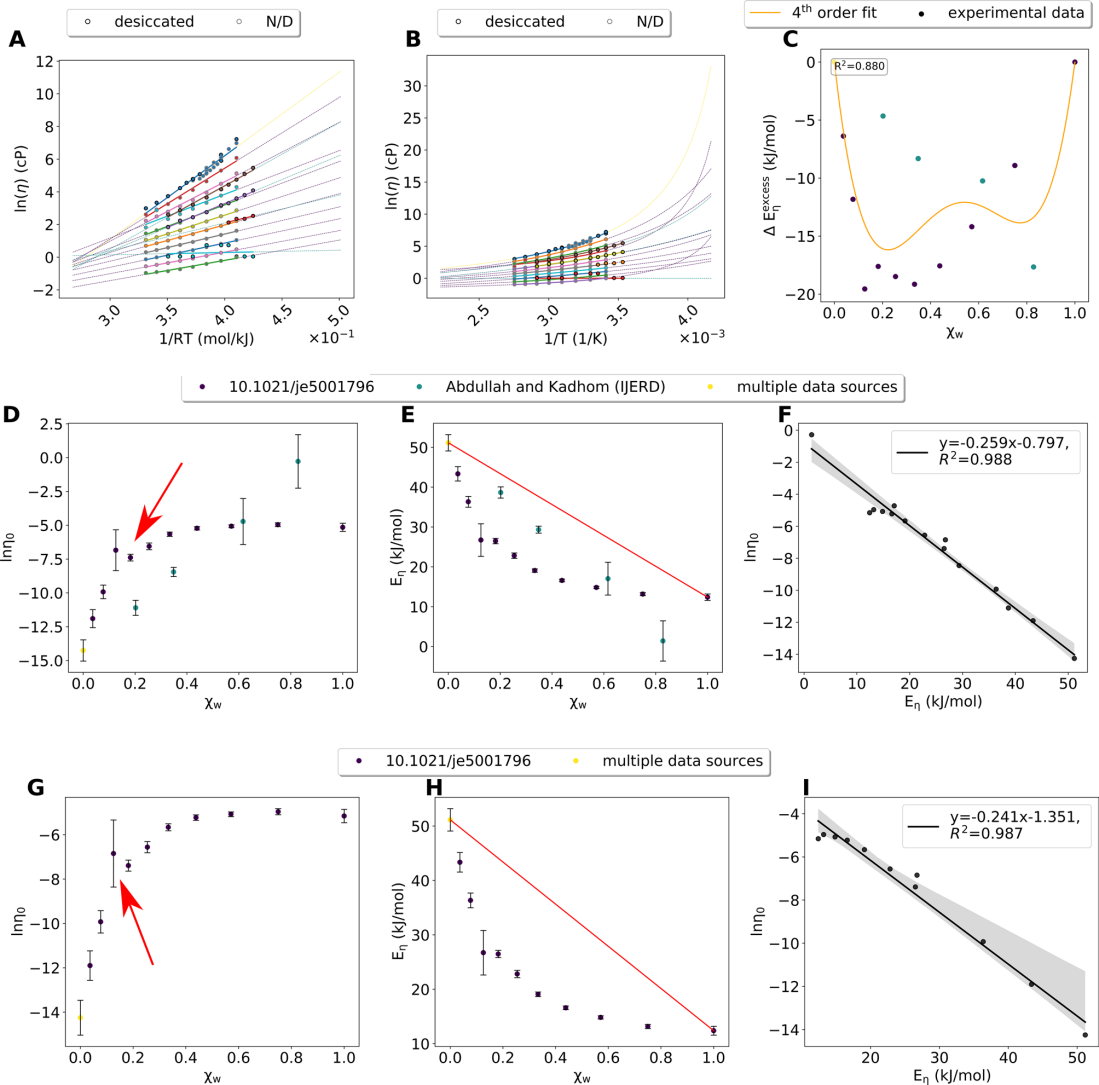
Dubious quality data for reline-water mixtures. (**A**) Arrhenius fits (using a minimum of 3 data points). Dots and thick lines are the experimental data and the respective fit. The dashed lines are extensions of the fit. Colors of the dashed lines indicate the source of the data. Yellow means multiple data points from different sources were be combined. (**B**) VFT fits (using a minimum of 4 data points). (**C**) ${E}^{excess}_{\eta}$, calculated based on the red line in (**E**). Colors of the data points indicate the source of the data. (**D**) ln*η*_*0*_ at different χw. Error bars are calculated based on the fit in (**A**).The red arrow highlights the data point affected by a presumed typo. Colors of the dashed lines indicate the source of the data. Yellow means multiple data points from different sources were be combined. (**E**) E_*η*_ at different χ_*w*_. The red line indicates the behavior of an ideal binary mixture and was used to calculate ${E}^{excess}_{\eta}$ (**C**). (**F**) Correlation between ln*η*_*0*_ and Eη. (**G**–**I**): same as (**D**–**F**), but without data from the source in a predatory journal, but with the data point affected by a presumed typo (red arrow in **G**).

A comprehensive analysis of data from different sources is pivotal for assessing the quality of individual data sources. For reline and glyceline–water mixtures, data retrieved from a source in a predatory journal^53^ (as per these lists: https://beallslist.net/ and https://predatoryjournals.com/journals/#I) behaved completely different from data from other sources (Figs. 1, 2 vs Fig. 3 and Supplementary Figure S18A–F). This data also deviated from the other data in the Arrhenius fits (Fig. 3A), resulting in a linear rather than a convex dependency of ln*η*_*0*_ (*χ*_*w*_) and *E*_*η*_(*χ*_*w*_) (Fig. 3D,E) and inconclusive values of ${E}^{excess}_{\eta}$ (*χ*_*w*_) and correlations of ln*η*_*0*_ and *E*_*η*_ (Fig. 3C,F). Despite the fact that the authors reported the desiccation method (Fig. 3A,B), we excluded this dataset from our analysis. For an automated analysis of large datasets, the quality and consistency of each data point matters. Each data point must have an associated error, which was only the case for half the data collected. Single outliers from dubious sources or corrupted by a typo might result in large uncertainties of ln*η*_*0*_ and *E*_*η*_ values as demonstrated for reline (Fig. 3G–I). To ensure data quality, typos should be prevented by applying the 4-eyes-principle, by data visualization prior to publication, or by using an electronic laboratory notebook^54^ for an automated data recording and a machine-readable data format such as CML^48^.

The comprehensive analysis of data from different sources also enabled us to compare the performance of two different phenomenological models, Arrhenius and VFT, in analyzing the data. Because of the slight convexity of the ln*η−*1/T curves, the VFT model was superior to the Arrhenius model in fitting viscosity data over the complete temperature range. However, the derived parameters A, B, and T_0_ showed an irregular dependency on *χ*_*w*_, and a general trend as for the parameters ln*η*_*0*_ and *E*_*η*_ from the Arrhenius model was not observed, as reported previously^15^. In the measured temperature range, the parameters of the VFT model are partially correlated^55^, or the model developed to describe the viscosity of glasses cannot be applied to aqueous DES mixture.

The systematic, comprehensive analysis of experimental viscosity data enabled a deep insight into the relationship between temperature and viscosity of aqueous mixtures. In the Arrhenius model, the two parameters *E*_*η*_ and ln*η*_*0*_ describe the temperature dependent and the temperature independent contributions, respectively, to viscosity. In the reported temperature range between 280 and 360 K, the temperature-dependent contribution dominates. The large value of *E*_*η*_ at low *χ*_*w*_ for all aqueous mixtures (except for methanol, Supplementary Figure S19A,B) indicates an increasing temperature sensitivity at decreasing water content. The choice of the hydrogen bond donor (glycerol, urea, or ethylene glycol) impacts the temperature dependency *E*_*η*_ of the viscosity. Urea increases *E*_*η*_ as compared to glycerol (51.2 and 42.3 kJ/mol for pure reline and glyceline, respectively), while ethylene glycol decreases *E*_*η*_ (46.6 and 30.4 kJ/mol for pure DEACG and DEACEG, respectively). In contrast, the salt had a minor effect, as pure glyceline and pure DEACG had comparable temperature dependencies (*E*_*η*_ of 42.3 and 46.6 kJ/mol, respectively).

Even more surprising was the observed relationship between water content and viscosity, obtained by the broad coverage of parameter space (different components, water content, and temperatures). Despite their difference in size, structure, polarity, and viscosity, the aqueous mixtures of three alcohols (ethylene glycol, methanol, glycerol) and three DESs (DEACEG, DEAG, glyceline) had a similar deviation ${E}^{excess}_{\eta}$*(χ*_*w*_) from ideal mixtures. It was similar for glycerol and methanol, despite the considerable difference of their viscosities (1412 cP and 0.585 cP, respectively, at 293.15 K for the pure compound), which was higher or lower, respectively, than pure water (1.002 cP at 293.15 K). The positive value of ${E}^{excess}_{\eta}$ is in agreement with a previous study, which reported that the addition of methanol to pure water resulted in a gradual decrease of the self-diffusion coefficients of both water and methanol, despite the fact that the self-diffusion coefficient of pure methanol is higher than of pure water^56^. Molecular dynamics simulations identified a possible reason of this excess behavior: the addition of the hydrophobic methyl group weakened the hydrogen bonding of water, whereas the hydroxyl group did not compensate for the loss of hydrogen bonds^57^. At increasing methanol concentrations, the diffusion of methanol further decreased by the formation of methanol clusters of increasing size, until at *χ*_*w*_ = 0.5–0.6 the system-wide water network broke down and the trend was reversed. Interestingly, all investigated aqueous mixtures showed a similar dependency ${E}^{excess}_{\eta}$*(χ*_*w*_), except for reline. The strongly non-ideal mixing behavior of the viscosity and the highly negative values of ${E}^{excess}_{\eta}$ of aqueous reline mixtures are surprising, because the densities of aqueous reline mixtures decrease almost linearly with water content (Supplementary Figure S20)^48^. However, it can be explained by the observation that, in contrast to aliphatic alcohols, the addition of urea to water has a negligible effect on the hydrogen-bond network of water at *χ*_*w*_ > 0.8^51,58^. Therefore, despite its higher viscosity, addition of reline to water barely increases the viscosity of the aqueous reline mixture, resulting in the highly negative ${E}^{excess}_{\eta}$.

## Conclusion

In this study, published experimental data on the temperature dependency of viscosity of different aqueous DES mixtures was systematically collected. The comprehensive analysis of the data resulted in two major observations: (1) aqueous reline mixtures differ fundamentally from all other DES. At increasing water content, their excess activation energy of viscous flow is negative, whereas it is positive for all other aqueous DES mixtures. (2) Experimental data as reported by different research groups might deviate considerably. Due to poor reporting of experimental methodologies, it is often impossible to identify the reason for the observed deviations. In order to make experiments reproducible, data and metadata have to be reported according to the F.A.I.R. principles. Access to open and structured data enables systematic meta-analyses and provides a deeper insight into the thermophysical properties of DES.

Our approach to collect and analyze thermophysical properties can also be applied to other solvents mixtures. Notably, DES with varying molar ratios could be studied to determine the impact of this parameter on the viscosity.

All data is available in a machine- and human readable format, the Chemical Markup Language (CML).

## Methods

### Data collection

Viscosity data for the aqueous solutions of two DES-salts choline chloride (ChCl) and *N*,*N*-diethylethanol ammonium chloride (DAC) and three DES-hydrogen bond donors (urea, glycerol, and ethylene glycol), and the resulting aqueous mixtures of DES were collected. We have also included water and methanol–water mixtures. Scientific publications containing data were searched for with the google scholar search tool. Keywords used were “DESs” (only for DES), “aqueous solution”, “viscosity”, and the name of the mixture [ChCl, DAC, urea, glycerol, ethylene glycol, reline, glyceline, DAC-glycerol (DEACG), DAC-ethylene glycol (DEACEG), methanol or water]. Data was extracted from tables where possible, and if only plots were available, data was extracted using PlotDigitizer (version 2.6.8).

Most of the published datasets on aqueous mixtures also included the viscosity of pure water (χ_w_ = 1.0). These data points were analyzed separately and compared to viscosity data for pure water from two other sources^35,36^. The workflow used for handling, analyzing, and plotting data is available on FAIRDOMHub (10.15490/FAIRDOMHUB.1.STUDY.767.1). All data sources are referenced by their DOI in the CML file.

### Parameters

The viscosity of the studied DES mixtures depends on the molar ratio of the DES-components (*r*_*DES*_, in mol/mol, Eq. 1), the water content (*χ*_*w*_, in mol/mol, Eq. 2) and the temperature (*T*).

1 $${r}_{DES} =\frac{{n}_{salt }}{{n}_{HBD}}$$

2 $${\upchi }_{w} =\frac{{n}_{water}}{{n}_{water}+{n}_{salt}+{n}_{HBD}}$$

with *n*_*salt*_, *n*_*HBD*_, and *n*_*water*_ denoting the relative number of ion pairs, hydrogen bond donor molecules, and water molecules in a mixture.

For binary aqueous solutions of the DES components and methanol, only *χ*_*w*_ and *T* are relevant, and *r*_*DES*_ is set to 0:

3 $${\upchi }_{w} =\frac{{n}_{water}}{{n}_{water}+{n}_{component}}$$

Two phenomenological models were applied to fit the temperature dependency of viscosity: the Arrhenius model and the Vogel–Fulcher–Tammann–Hesse (VFT) model.

### Arrhenius model

Only datasets for which at least three different temperatures were available were analyzed. The Arrhenius model assumes a linear relationship between *lnη* and T^−1^:

4 $$ln =\mathrm{ln}{}_{0}+ \frac{E}{RT}$$

with the activation energy of viscous flow *E*_*η*_ (in kJ/mol) and the viscosity at infinite temperature *η*_*0*_ as parameters.

For ideal binary mixtures, *lnη* is additive and therefore *E*_*η*_ and *lnη*_*0*_ are linear in *χ*_*1*_^59^:

5a $${\text{E}}_{\eta } = \chi_{{1}} \times {\text{E}}_{{\eta {1}}} + \chi_{{2}} \times {\text{E}}_{{\eta {2}}} = \chi_{{1}} \times ({\text{E}}_{{\eta {1}}} - {\text{E}}_{{\eta {2}}} ) \, + {\text{ E}}_{{\eta {2}}}$$

5b $${\text{ln}}\eta_{0} = \chi_{{1}} \times {\text{ln}}\eta_{01} + \chi_{{2}} \times {\text{ln}}\eta_{02} = \chi_{{1}} \times ({\text{ln}}\eta_{01} - {\text{ ln}}\eta_{02} ) \, + {\text{ ln}}\eta_{02}$$

where *χ*_*1*_ and *χ*_*2*_ are the mole fractions of the two components of the binary mixture (*χ*_*1*_ + *χ*_*2*_ = *1*), *E*_*η1*_ and *E*_*η2*_ the respective activation energies, *η*_*01*_ and *η*_*02*_ the respective viscosities at infinite temperature.

${E}^{excess}_{\eta}$ was calculated as the deviation of *E*_*η*_ from an ideal mixture by fitting a linear regression through *E*_*η*_ at *χ*_*w*_ = 0 and *χ*_*w*_ = 1. ${E}^{excess}_{\eta}$ was fitted by polynomials of 4th order, biased by forcing the fit through the most extreme data points (e.g. ${E}^{excess}_{\eta}$=0 at *χ*_*w*_ = 0 and *χ*_*w*_ = 1).

For pure liquids at *χ*_*w*_ = 0 and *χ*_*w*_ = 1, the temperature dependency of viscosity *η* is described by an Arrhenius equation:

6a $${\text{ln}}\eta (T,\chi_{w} = 0) = {\text{E}}_{\eta } (\chi_{{\text{w}}} = 0)/{\text{RT}} + {\text{ln}}\eta_{0} (\chi_{w} = 0)$$

6b $${\text{ln}}\eta (T,\chi_{w} = {1}) = {\text{ E}}_{\eta } (\chi_{w} = {1}) /{\text{RT }} + {\text{ ln}}\eta_{0} (\chi_{w} = {1})$$

Thus, there is a temperature *T*_*η*_ at which

7a $${\text{ln}}\eta (T_{\eta } ,\chi_{w} = 0) = {\text{ ln}}\eta (T_{\eta } ,\chi_{w} = {1})$$

with

7b $$RT_{\eta } = (E_{\eta } (\chi_{w} = 0) - E_{\eta } (\chi_{{\text{w}}} = {1}))/({\text{ln}}\eta_{0} (\chi_{w} = {1}) - {\text{ln}}\eta_{0} (\chi_{w} = 0))$$

Assuming ideal mixing, all mixtures *χ*_*w*_ = 0…1 will have the same viscosity ln*η*(*T*_*η*_), thus ln*η*(*T*_*η*_) is independent of *χ*_*w*_ for all *χ*_*w*_ = 0…1:

8a $${\text{ln}}\eta (T_{\eta } ) = E_{\eta } (\chi_{w} )/RT_{\eta } + {\text{ln}}\eta_{0} (\chi_{w} )$$

This independence results in a linear correlation between *E*_*η*_(*χ*_*w*_) and ln*η*_*0*_(*χ*_*w*_):

8b $${\text{ln}}\eta_{0} (\chi_{w} ) = - (RT_{\eta } )^{{ - {1}}} \times E_{\eta } (\chi_{w} ) + {\text{ln}}\eta (T_{\eta } )$$

with a slope -(*RT*_*η*_)^-1^ and intercept with the y-axis at ln*η*(*T*_*η*_).

A deviation from ideal mixing has two consequences:

1. Not all curves ln*η*(*T, χ*_*w*_) will intersect at *T* = *T*_*η*_ (Eq. 8a)
2. There will be deviations from the linear correlation (Eq. 8b)

For non-ideal mixtures, *E*_*η*_(*χ*_*w*_) deviates from its ideal value *E*_*η*_^*ideal*^(*χ*_*w*_) by ${E}^{excess}_{\eta}$ (*χ*_*w*_) :

9 $$E_{\eta } (\chi_{w} ) = E_{\eta }^{ideal} (\chi_{w} ) + E_{\eta }^{excess} (\chi_{w} )$$

with

10 $$E_{\eta }^{ideal} (\chi_{w} ) = (1 - \chi_{w} ) \times E_{\eta } (\chi_{w} = 0) + \chi_{w} \times E_{\eta } (\chi_{w} = 1)$$

Because ln*η*_*0*_*(χ*_*w*_*)* depends on *E*_*η*_*(χ*_*w*_*)* according to (Eq. 8b), *lnη(T, χ*_*w*_*)* of a binary mixture can be predicted by determining by experiment or by simulation:

1. *E*_*η*_ and *lnη*_*0*_ of the two pure components (*χ*_*w*_ = 0 and *χ*_*w*_ = 1)
2. ${E}^{excess}_{\eta}$* (*χ_*w*_*)* of the binary mixtures

### Vogel–Fulcher–Tammann–Hesse model

Only datasets for which at least four different temperatures were available were analyzed. The Vogel–Fulcher–Tammann–Hesse (VFT) model (Eq. 11) was developed to describe the temperature dependency of viscosity^10–12^ and can be applied to ionic liquids^60–62^.

11 $$ln =A+ \frac{B}{T-{T}_{0}}$$

The empirical constants *A*, *B,* and *T*_*0*_ were determined using initial parameters derived from Yadav et al*.* (A = − 2, B = 800, *T*_*0*_ = 170 K)^15^.

### Data quality

The data sets were manually curated and checked to eliminate copy-paste errors. A recurring issue was the use of “,” or “.” as a symbol for the decimal point when using the “German-language-Microsoft-Excel”. This issue lead to 0,809 (instead of 0.809) to become 809, when the csv file was opened in Excel. A further complication was the use of different units (e.g. mP or cP). One data point from 10.1021/je5001796 was removed because it was assumed to be a typing error (*η* = 17.742 cP at *r*_*DES*_ = 0.5, *T* = 353.15 K*, χ*_*w*_ = 0.126, see Discussion, Supplementary Figure S10, S18). Data from a source in a predatory journal^53^ (as per these lists: https://beallslist.net/ and https://predatoryjournals.com/journals/#I) was also removed (see “Discussion”, Supplementary Figs. S18. S19).

### Chemical markup language

The chemical markup language (CML) was used to integrate the viscosity data retrieved from literature. The data was copied manually from literature into csv files, which were then converted to CML using Python scripts as previously described. The CML concepts were defined using the CompChem Convention^63^ to describe mixtures and their viscosities, the origin of the data (experiment), data properties (DOI, ID, value, error), and parameters (molar ratio of DES, mole fraction of water, and temperature). As previously described, the CML data was then analyzed and visualized using Python scripts^48^.

### Workflow used for analysis

The analysis scripts are organized in a workflow which requires the user to modify the names.py script and run the wrapper.py script. Names.py contains the name of files and parameters that will be analyzed with the workflow. Data can be filtered using the variables ‘quality’, ‘variables’ and ‘myfilters’. Wrapper.py will import all the required functionalities from the provided scripts. Details can be found in section 2 in Supplementary Information (“Instructions for using the workflow”).

XML files were written and parsed with ﻿xml.etree.cElementTree^64^. The values of *E*_*η*_ and ln*η*_*0*_ and their error estimates were obtained by the curve_fit function from Scipy^65^. The fitting of excess Eη was achieved through numpy Polynomial module^66^. The figures were visualized by python modules ﻿matplotlib.pyplot^67^ and library seaborn^69^. Additional python libraries used were pandas^69^, sys^70^, os^71^, subprocess^72^.

## Supplementary information

Supplementary Information.
