# Supplementary material for: Meta-analysis of viscosity of aqueous deep eutectic solvents and their components
Source: Sci Rep. 2020 Dec 7;10:21395. doi: 10.1038/s41598-020-78101-y (PMC7721810; doi:10.1038/s41598-020-78101-y)
Supplement: Supplementary file 1 — Supplementary Information. [file 41598_2020_78101_MOESM1_ESM.pdf]

Supporting Information

Meta-analysis of viscosity of aqueous deep eutectic solvents and their components

Gudrun Gygli[1,§], Xinmeng Xu[2,§], Jürgen Pleiss[2,*]

[1] Institute for Biological Interfaces 1, Karlsruhe Institute of Technology, Eggenstein-Leopoldshafen, 76344 Germany

[2] Institute of Biochemistry and Technical Biochemistry, University of Stuttgart, Stuttgart, 70569, Germany

[§] Both authors contributed equally to this work

* Corresponding author:

Jürgen Pleiss

Institute of Biochemistry and Technical Biochemistry

University of Stuttgart

Allmandring 31

70569 Stuttgart, Germany

E-mail: Juergen.Pleiss@itb.uni-stuttgart.de

ORCID: 0000-0003-1045-8202

This document contains two sections, the supplementary figures and the instructions for using the analysis workflow. The figures and tables in the Instructions section are not referenced in the main text (Table I1, Figures I1-I2).

Supplementary Figures

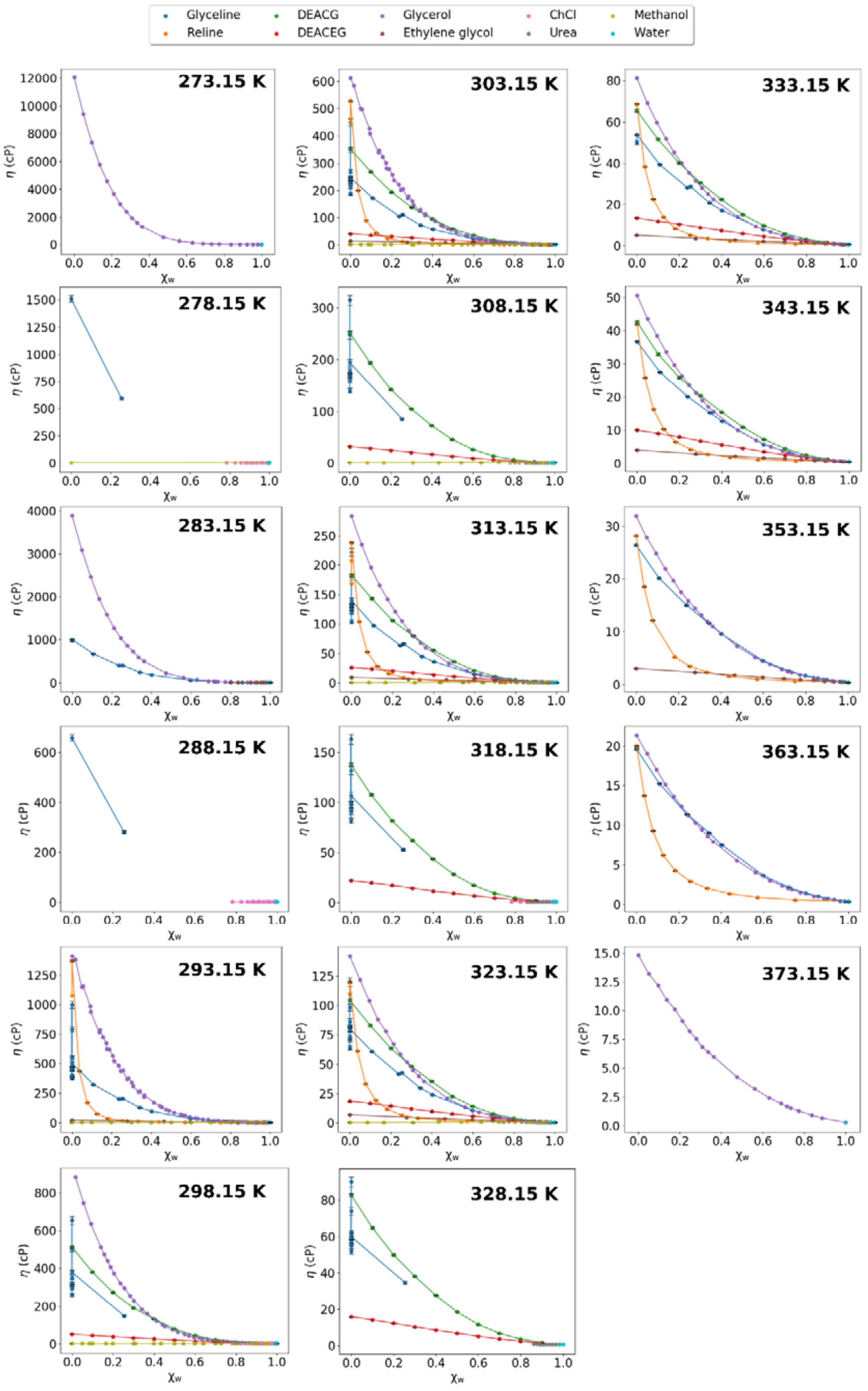

Figure S1: Overview of the water dependency (χ_w) of viscosity at different temperatures. The colors indicate the mixture. The lines are simply connections between the data points and are not based on any modelling.

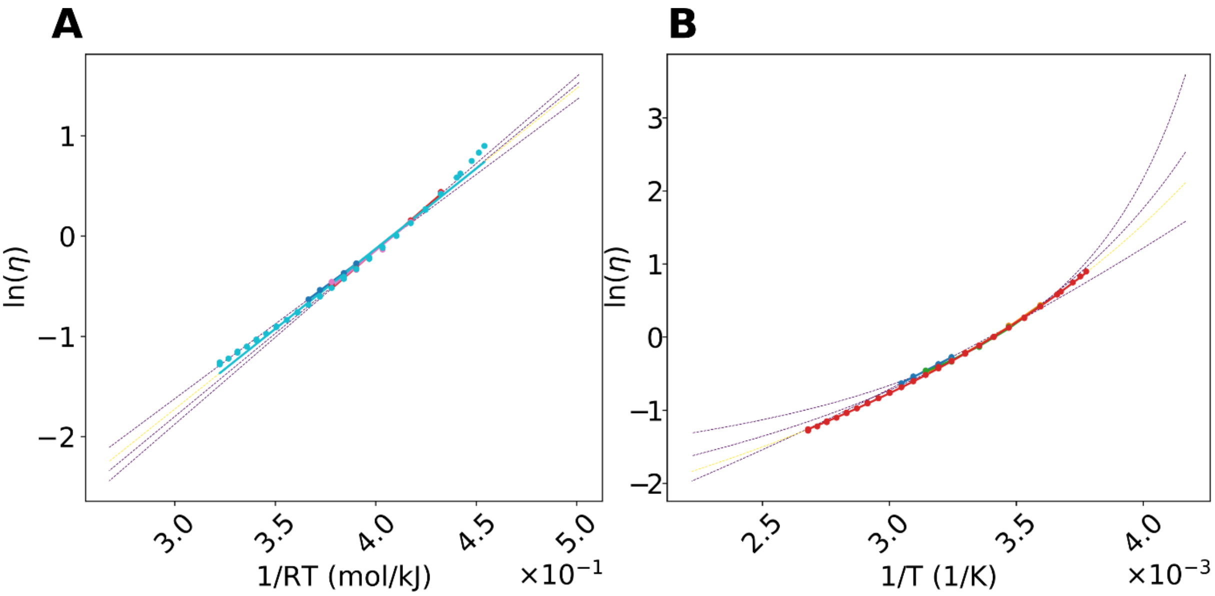

Figure S2: Pure water. A) Arrhenius fits (using a minimum of 3 data points). Dots and thick lines are the experimental data and the respective fit. The dashed lines are extensions of the fit to observe if an intersection of all fits exists. Colors of the dashed lines indicate the source of the data. Yellow means multiple data points from different sources were be combined. B) VFT fits (using a minimum of 4 data points) Same as A).

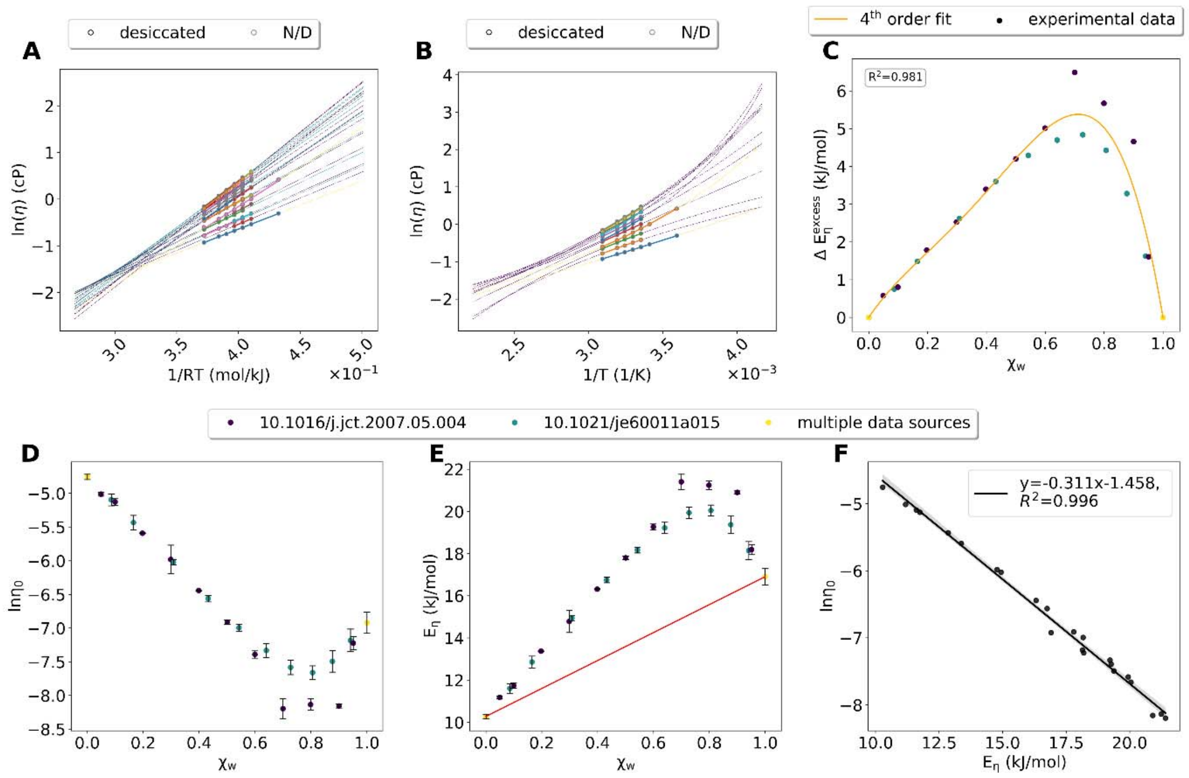

Figure S3: Methanol-water mixtures. A) Arrhenius fits (using a minimum of 3 data points). Dots and thick lines are the experimental data and the respective fit. The dashed lines are extensions of the fit. Colors of the dashed lines indicate the source of the data. Yellow means multiple data points from different sources were be combined. B) VFT fits (using a minimum of 4 data points. C) E_{η}^{excess}, calculated based on the red line in E. Colors of the data points indicate the source of the data. D) ln η_0 at different χ_w. Error bars are calculated based on the fit in A). Colors of the dashed lines indicate source of the data. E) E_η at different χ_w. The red line indicates the behavior of an ideal binary mixture and was used to calculate E_{η}^{excess} (C). F) Correlation between ln η_0 and E_η.

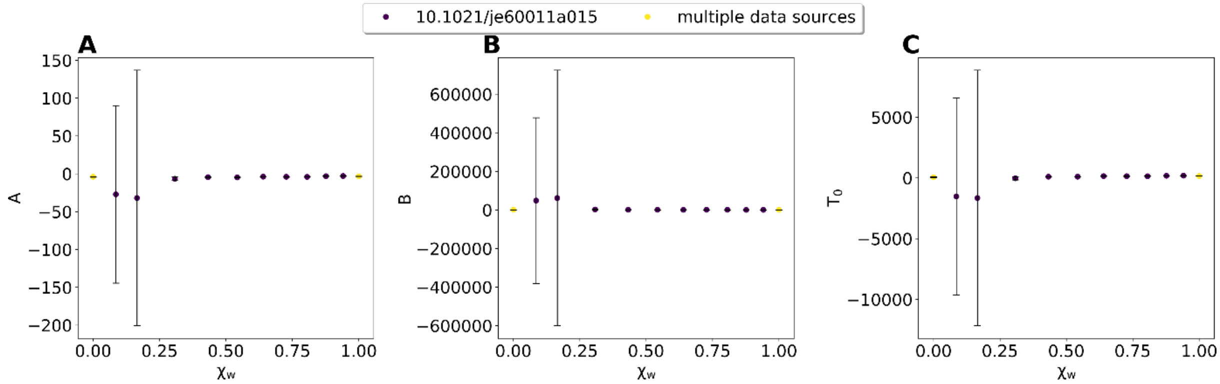

Figure S4: Water dependency (χ_w) of the VFT model parameters (A, B and T_0 in A-C, respectively) for methanol-water mixtures. The colors indicate source of the data. Yellow means multiple data points from different sources were be combined.

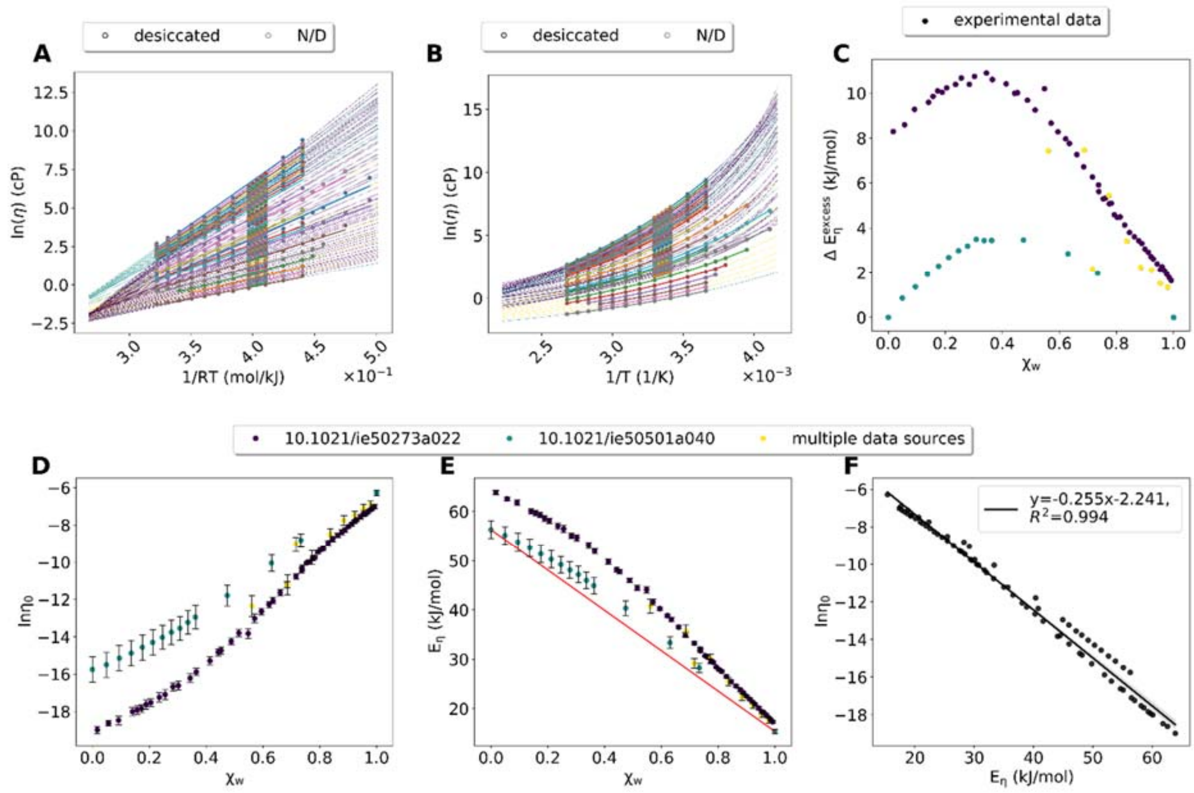

Figure S5: Glycerol-water mixtures. A) Arrhenius fits (using a minimum of 3 data points). Dots and thick lines are the experimental data and the respective fit. The dashed lines are extensions of the fit. Colors of the dashed lines indicate the source of the data. Yellow means multiple data points from different sources were be combined. B) VFT fits (using a minimum of 4 data points). C) E_η^{excess}, calculated based on the red line in E. Colors of the data points indicate the source of the data. D) ln η_0 at different χ_w. Error bars are calculated based on the fit in A). Colors of the dashed lines indicate source of the data. E) E_η at different χ_w. The red line indicates the behavior of an ideal binary mixture and was used to calculate E_η^{excess} (C). F) Correlation between ln η_0 and E_η.

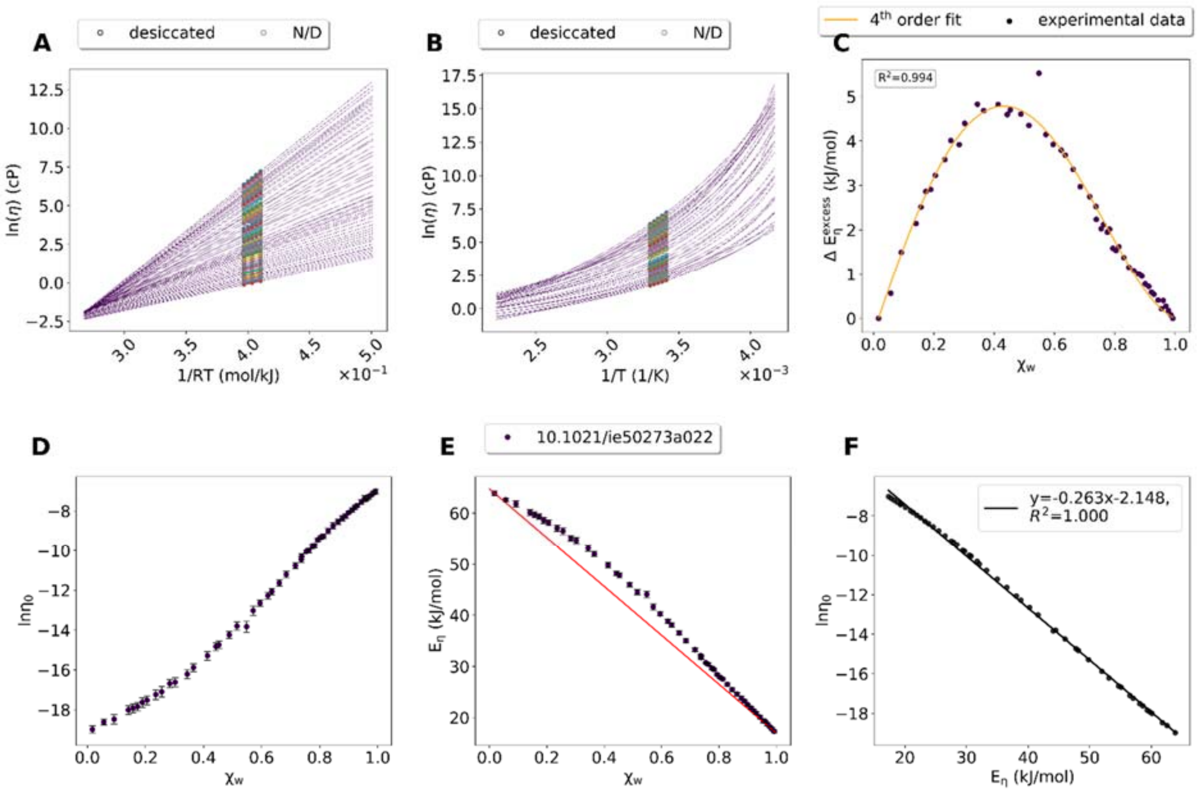

Figure S6: Glycerol-water mixtures, same as Figure S5, but with data from Sheely et al [28] only, allowing a fit in C.

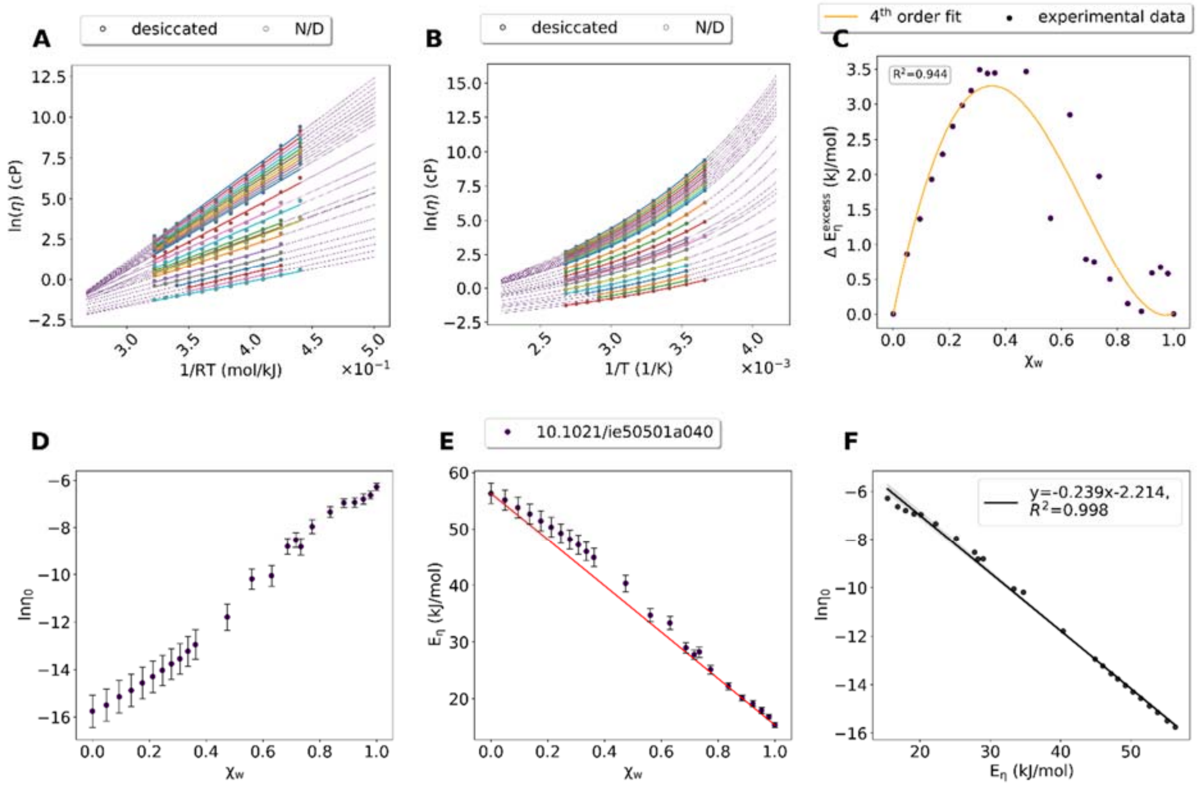

Figure S7: Glycerol-water mixtures, same as Figure S5, but with data from Segur et al [27] only, allowing a fit in C.

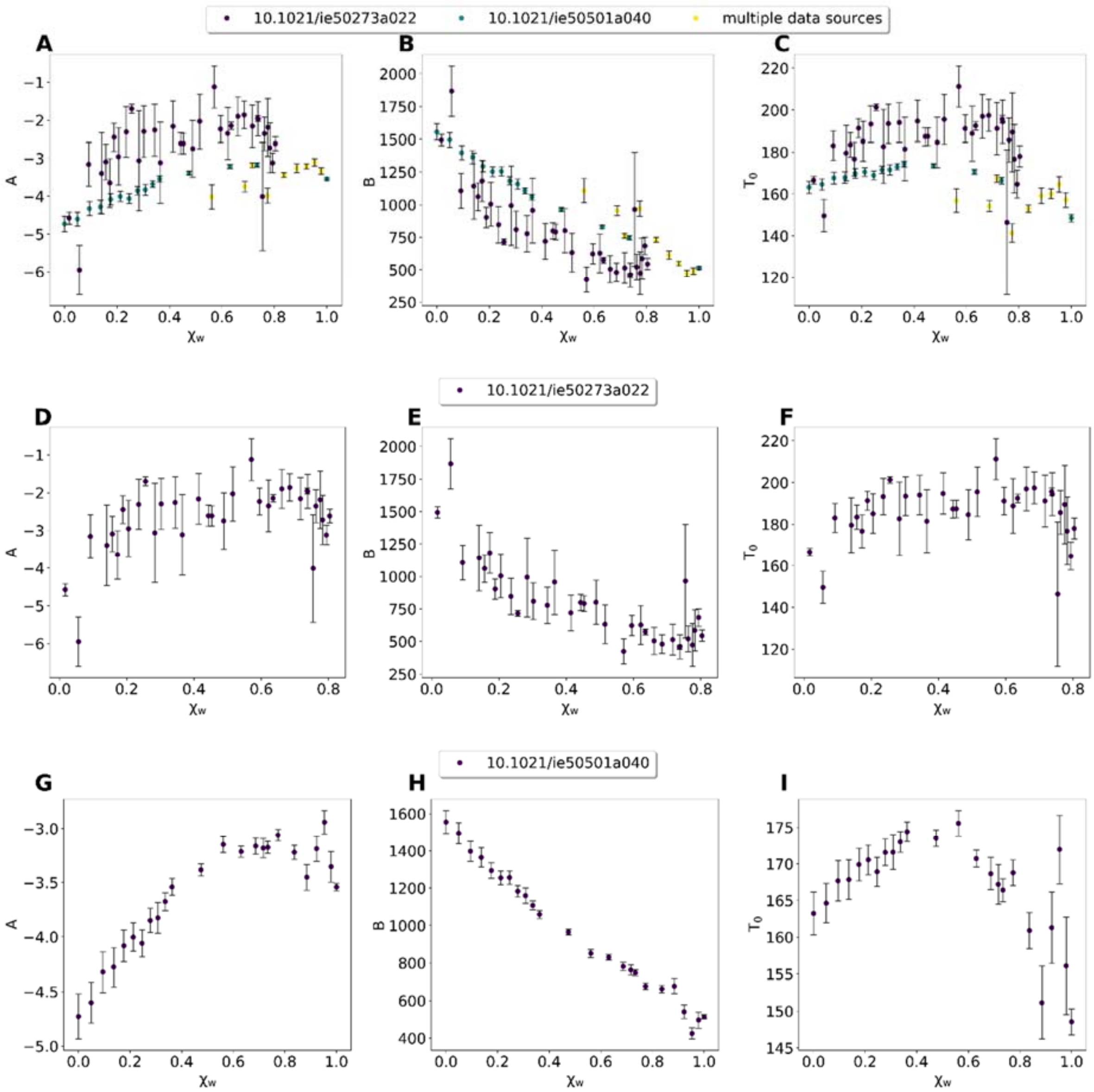

Figure S8: Water dependency (χ_w) of the VFT model parameters (A, B and T_0 in A-C, respectively) for glycerol-water mixtures. The colors indicate source of the data. Yellow means multiple data points from different sources were be combined. D-F same as A-C, but with data from Sheely et al [28] only. G-I same as A-C, but with data from Segur et al [27] only.

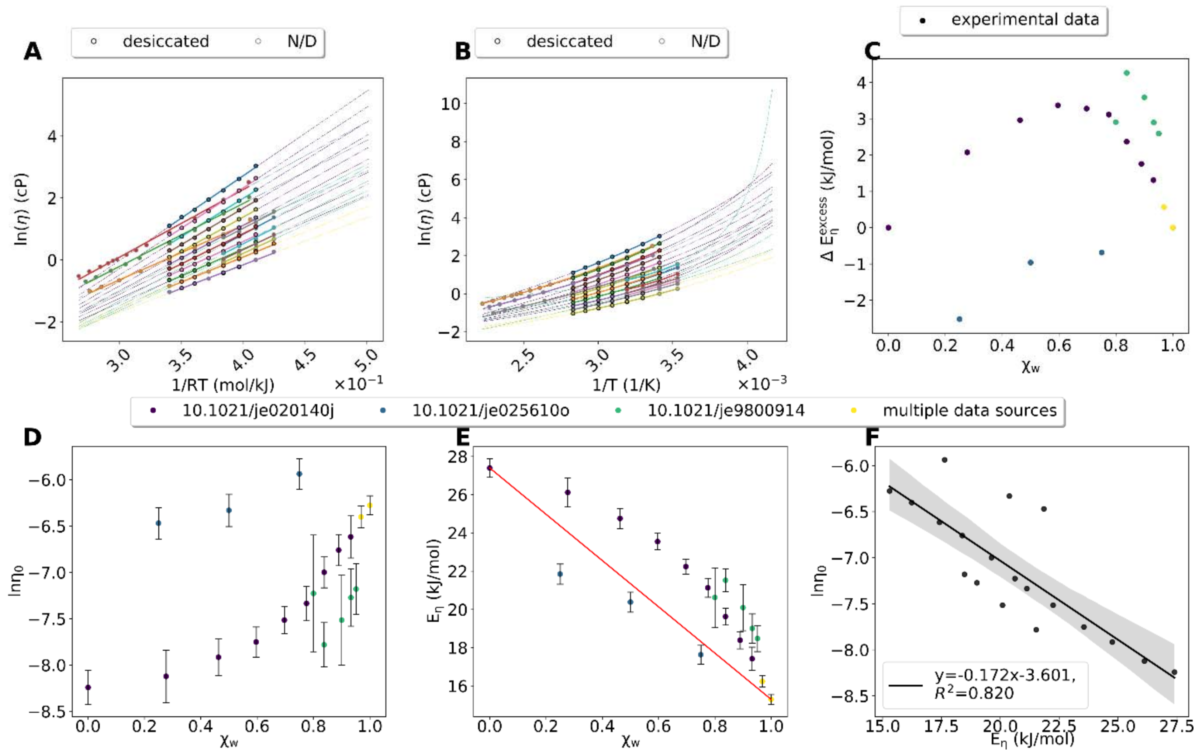

Figure S9: Ethylene glycol-water mixtures. A) Arrhenius fits (using a minimum of 3 data points). Dots and thick lines are the experimental data and the respective fit. The dashed lines are extensions of the fit. Colors of the dashed lines indicate the source of the data. Yellow means multiple data points from different sources were be combined. B) VFT fits (using a minimum of 4 data points). C) E_η^{excess}, calculated based on the red line in E. Colors of the data points indicate the source of the data. D) ln η_0 at different χ_w. Error bars are calculated based on the fit in A). Colors of the dashed lines indicate source of the data. E) E_η at different χ_w. The red line indicates the behavior of an ideal binary mixture and was used to calculate E_η^{excess}(C). F) Correlation between ln η_0 and E_η.

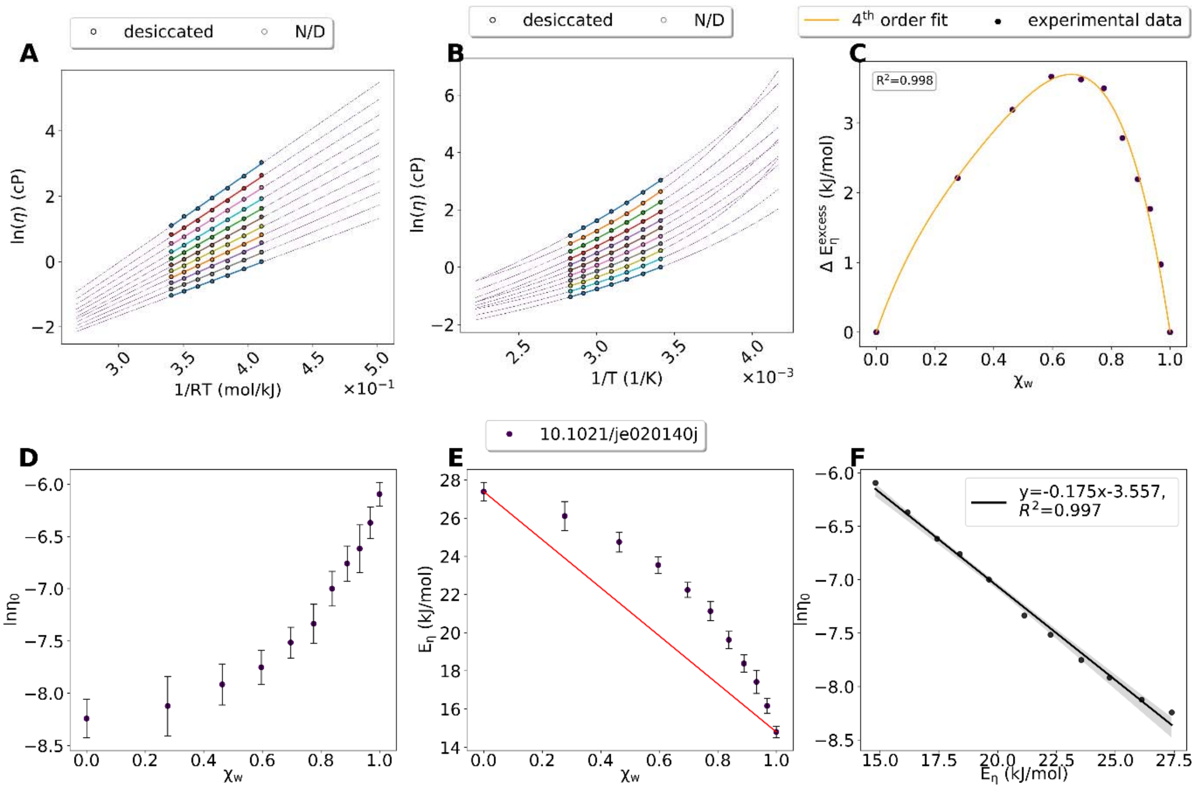

Figure S10: Ethylene glycol-water mixtures, same as Figure S9, but with data from Yang et al [24] *only, allowing a fit in C.*

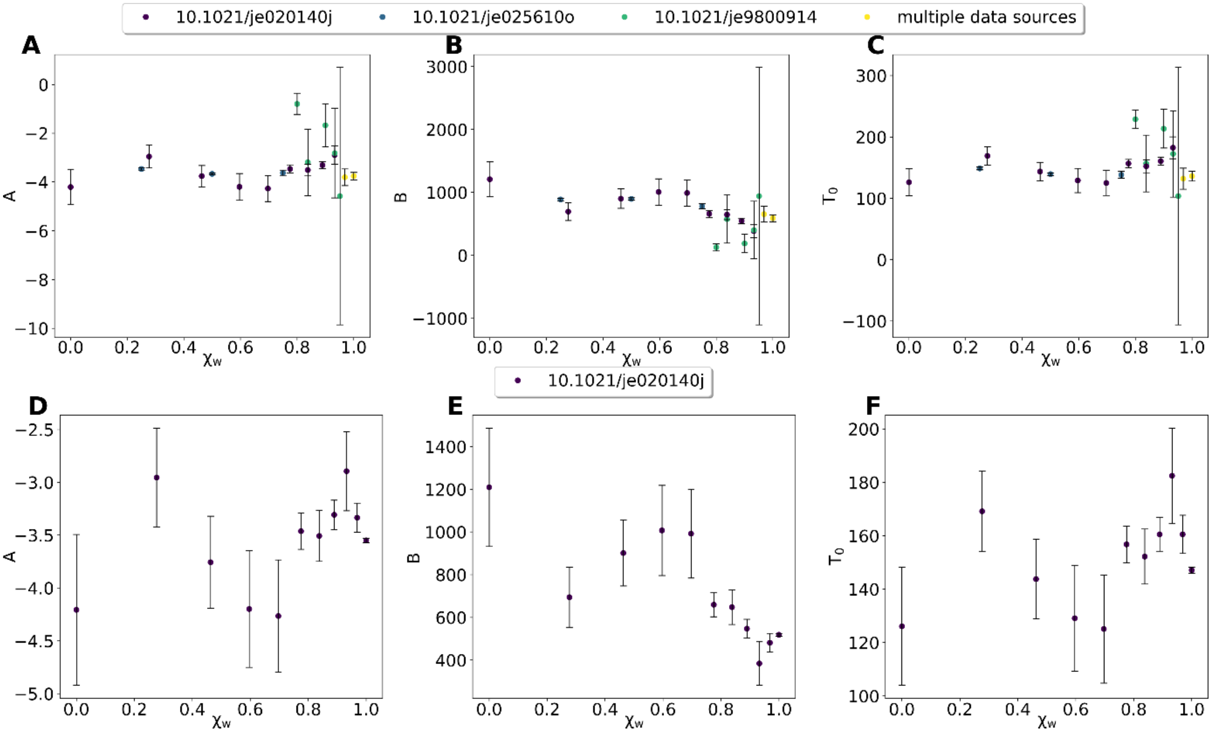

Figure S11: Water dependency (χ_w) of the VFT Model parameters (A, B and T_0 in A-C, respectively) for ethylene glycol-water mixtures. The colors indicate source of the data. Yellow means multiple data points from different sources were be combined. D-F same as A-C, but with data from Yang et al [24]only.

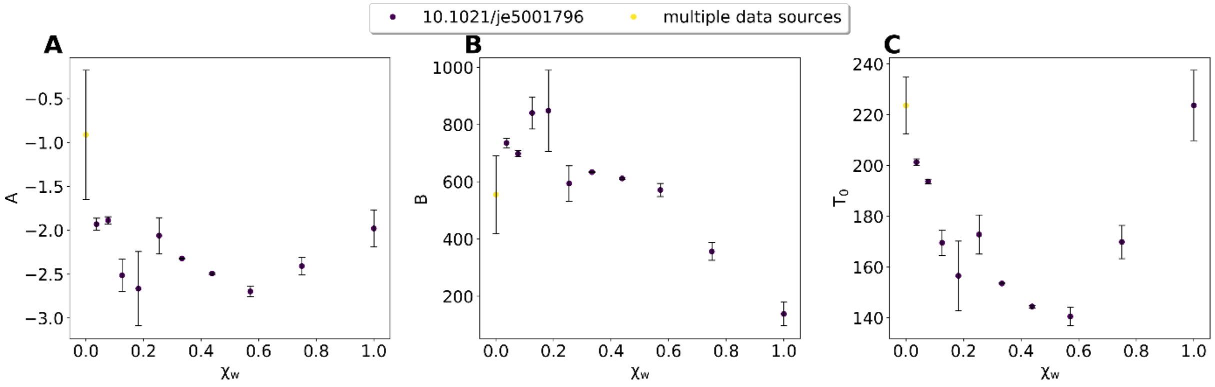

Figure S12: Water dependency (χ_w) of the VFT Model parameters (A, B and T_0 in A-C, respectively) for reline-water mixtures.

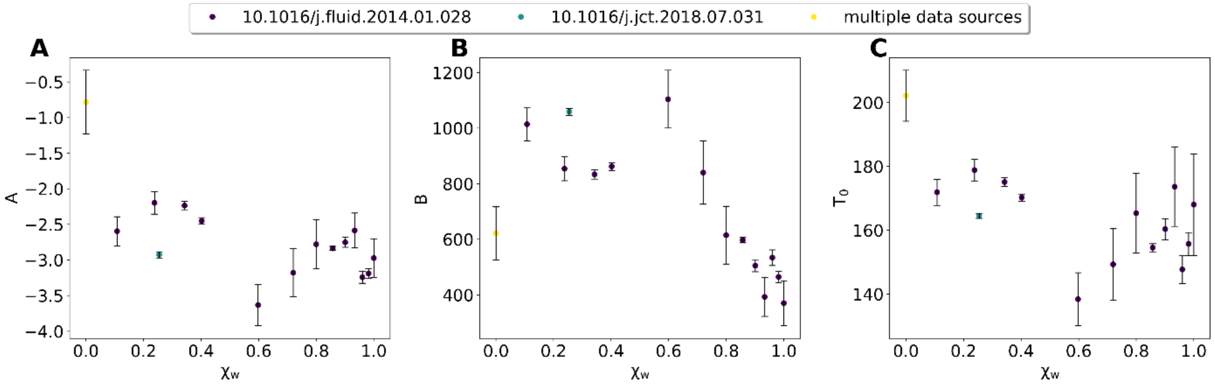

Figure S13: Water dependency (χ_w) of the VFT Model parameters (A, B and T_0 in A-C, respectively) for glyceline-water mixtures.

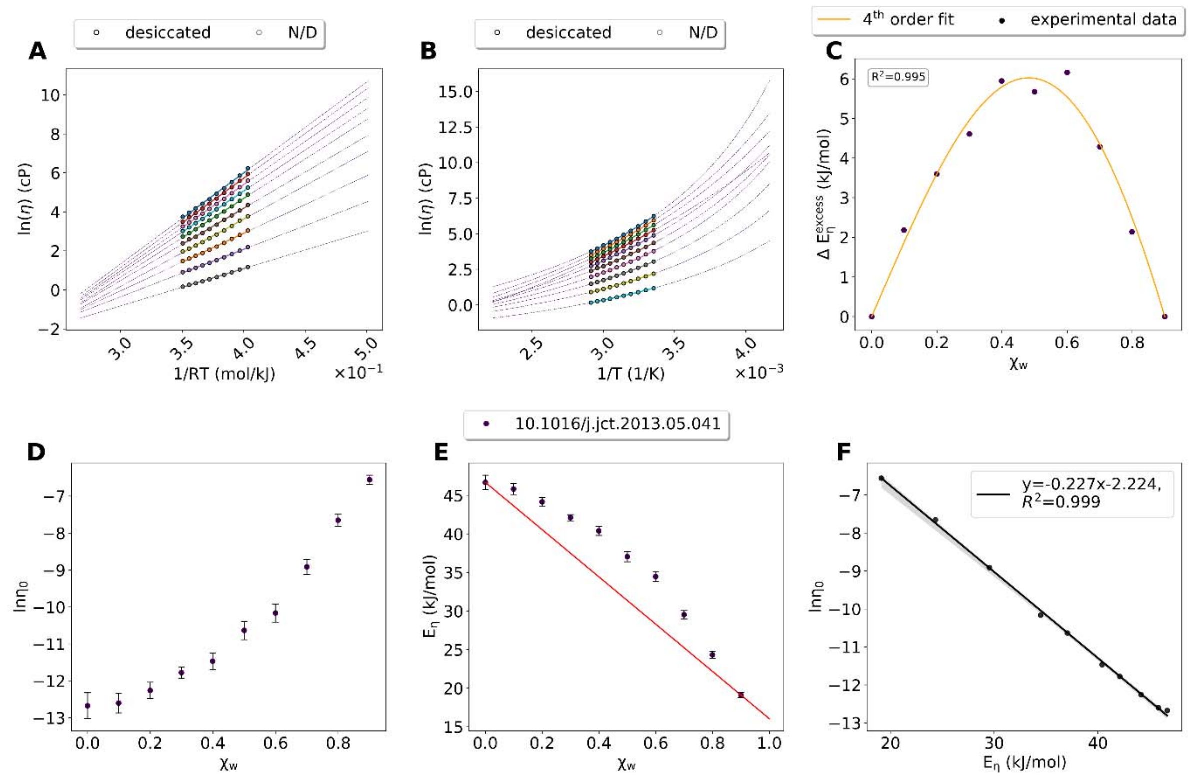

Figure S14: DEACG-water mixtures. A) Arrhenius fits (using a minimum of 3 data points). Dots and thick lines are the experimental data and the respective fit. The dashed lines are extensions of the fit. Colors of the dashed lines indicate the source of the data. Yellow means multiple data points from different sources were be combined. B) VFT fits (using a minimum of 4 data points. C) E_η^{excess}, calculated based on the red line in E. Colors of the data points indicate the source of the data. D) $\ln\eta_0$ at different χ_w. Error bars are calculated based on the fit in A). Colors of the dashed lines indicate source of the data. E) E_η at different χ_w. The red line indicates the behavior of an ideal binary mixture and was used to calculate E_η^{excess} (C). F) Correlation between $\ln\eta_0$ and E_η.

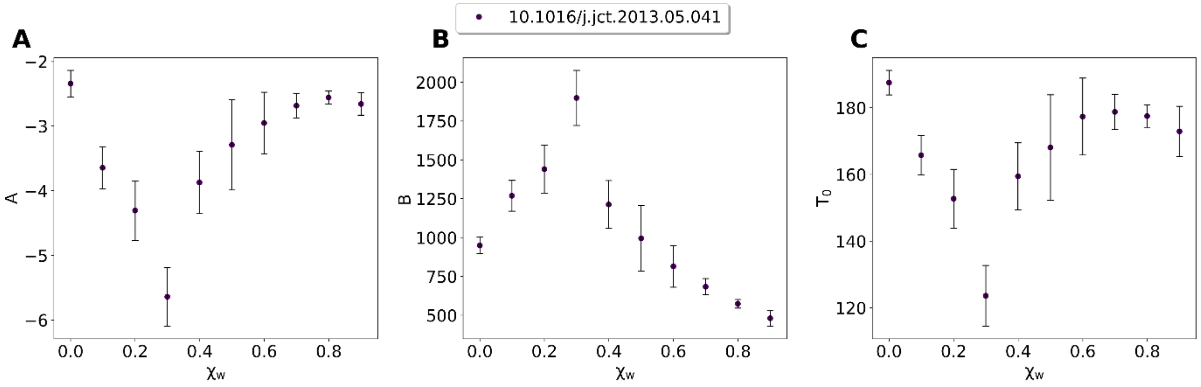

Figure S15: Water dependency (χ_w) of the VFT Model parameters (A, B and T_0 in A-C, respectively) for DEACG-water mixtures.

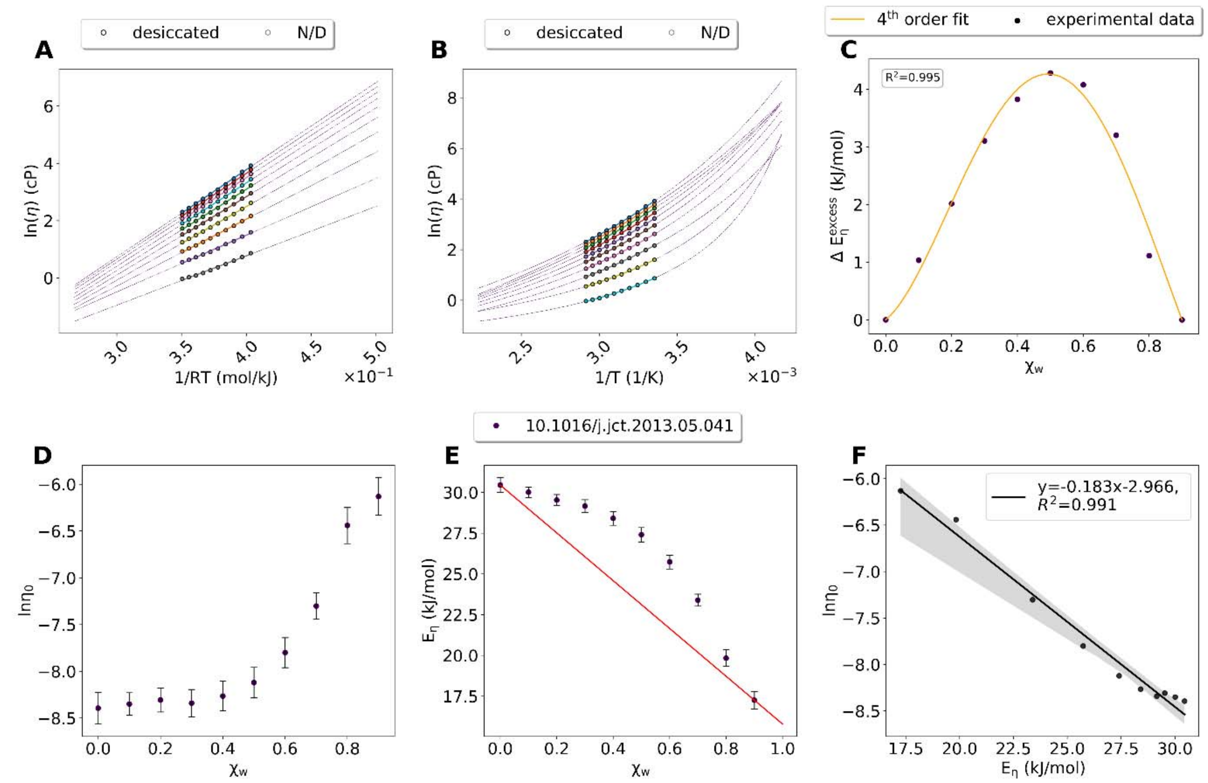

Figure S16: DEACEG-water mixtures. A) Arrhenius fits (using a minimum of 3 data points). Dots and thick lines are the experimental data and the respective fit. The dashed lines are extensions of the fit. Colors of the dashed lines indicate the source of the data. Yellow means multiple data points from different sources were be combined. B) VFT fits (using a minimum of 4 data points). C) E_{η}^{excess}, calculated based on the red line in E. Colors of the data points indicate the source of the data. D) $\ln\eta_0$ at different χ_w. Error bars are calculated based on the fit in A). Colors of the dashed lines indicate source of the data. E) E_{η} at different χ_w. The red line indicates the behavior of an ideal binary mixture and was used to calculate E_{η}^{excess} (C). F) Correlation between $\ln\eta_0$ and E_{η}.

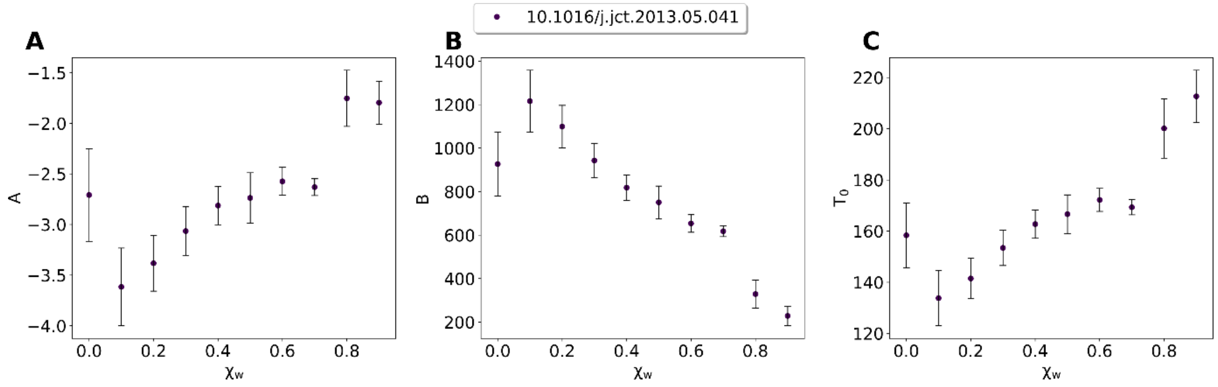

Figure S17: Water dependency (χ_w) of the VFT Model parameters (A, B and T_0 in A-C, respectively) for DEACEG-water mixtures.

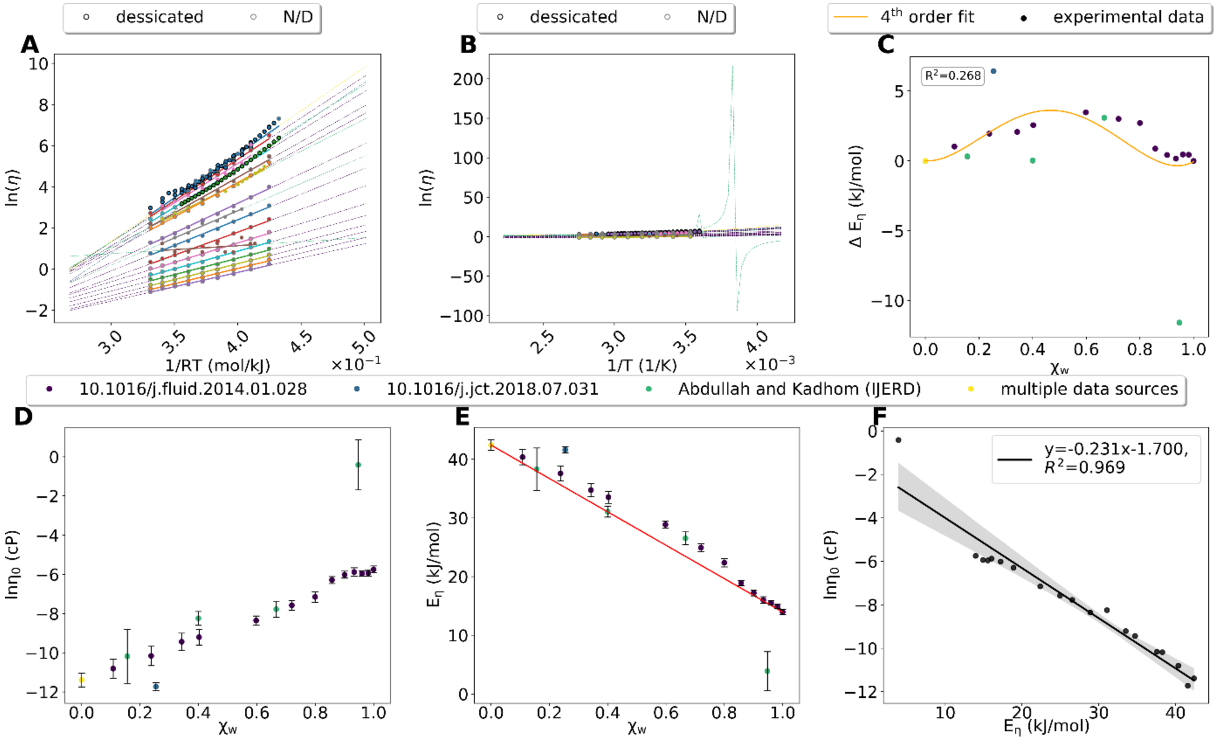

Figure 18: Dubious quality data for glyceline-water mixtures. A) Arrhenius fits (using a minimum of 3 data points). Dots and thick lines are the experimental data and the respective fit. The dashed lines are extensions of the fit. Colors of the dashed lines indicate the source of the data. Yellow means multiple data points from different sources were be combined. B) VFT fits (using a minimum of 4 data points). C) E_η^{excess}, calculated based on the red line in E. Colors of the data points indicate the source of the data. D) $\ln\eta_0$ at different χ_w. Error bars are calculated based on the fit in A). Colors of the dashed lines indicate source of the data. E) E_η at different χ_w. The red line indicates the behavior of an ideal binary mixture and was used to calculate E_η^{excess}(C). F) Correlation between $\ln\eta_0$ and E_η.

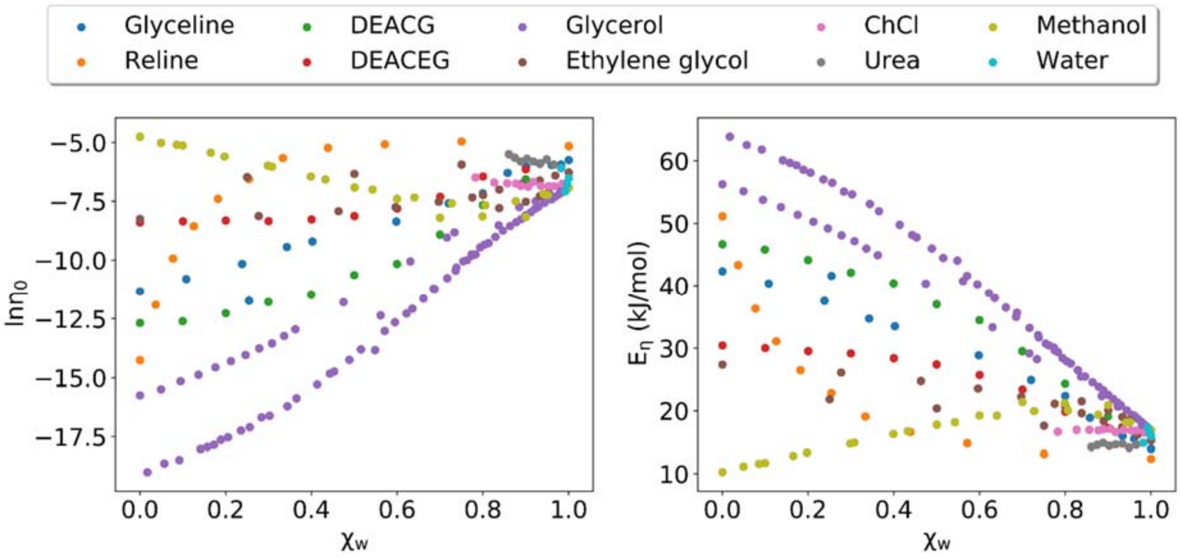

Figure S19: Overview of $ln\,\eta_0$ and E_η for direct comparison of the different mixtures analyzed. Note that the data is colored by mixture, and data from different sources is not separated here (e.g. the two data series for glycerol-water mixtures are colored the same).

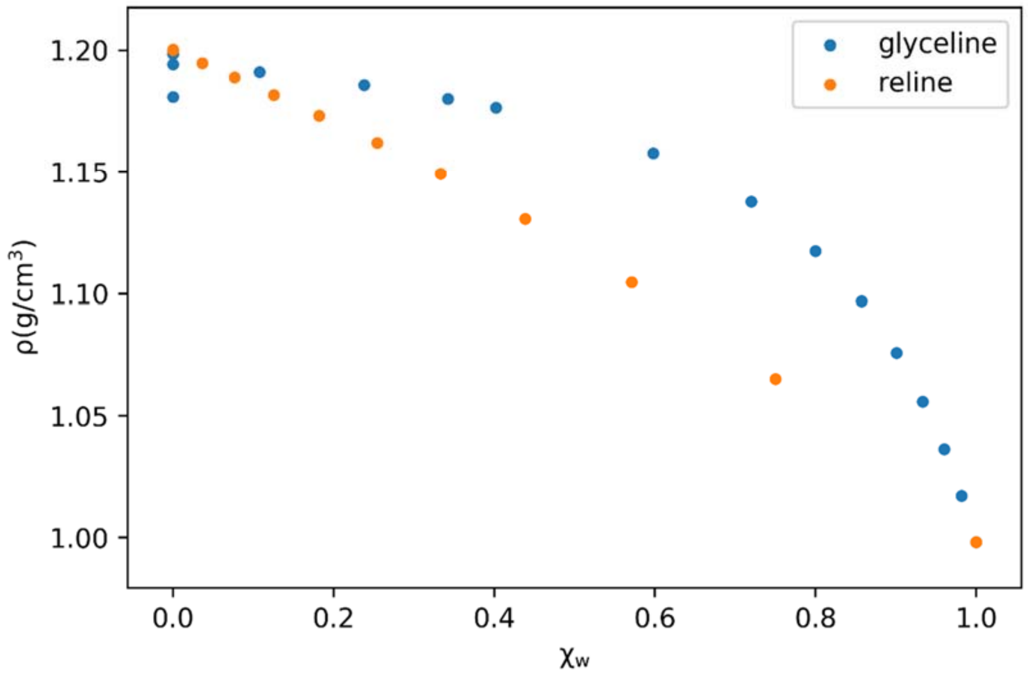

Figure S20: Densities at different χ_w of glyceline and reline at 303.15 K. Data from [48].

Instructions for using the Workflow

General remarks
The workflow is constructed with two main python scripts (“names.py” and “wrapper.py”) and imports functionalities from other scripts. “names.py” describes all information about mixture names and variables that are used to filter or analyze data. “wrapper.py” imports functions that were defined in five other python scripts located in the folder “Scripts”. The input csv files have to be located in the folder “Input” and be named in agreement with the mixture names used in “names.py”. “wrapper.py” extracts data from CSV files , writes them to CML files, filters, models (Arrhenius or VFT) and plots the data (Figures I1 and I2).

Names.py
In script “names.py”, the names of mixtures and names used in csv files are given (Table I1). The two variables to analyze are ‘ molar ratio of DES’ and ‘mole fraction of water’.

Table I1: This list shows the names of mixtures and their corresponding names used in the names of csv, CML files and png figures. First five names are deep eutectic solvents and the rest are names of aqueous solutions. Some csv files are named with extra information like ‘_DOI1’, ‘_withpredjournalpaper’.

mixture names	filenames
DES-Choline chloride glycerol	ChCl_glycerol
DES-Choline chloride ethylene glycol	ChCl_ethylene glycol
DES-Choline chloride urea	ChCl_urea
DES-N,N-diethylethanol ammonium chloride ethylene glycol	N,N-diethylethanol ammonium chloride_ethylene glycol
DES-N,N-diethylethanol ammonium chloride glycerol	N,N-diethylethanol ammonium chloride_glycerol
Aqueous sol. of ethylene glycol	ethylene glycol
Aqueous sol. of urea	urea
Aqueous sol. of Choline chloride	ChCl
Aqueous sol. of glycerol	glycerol

Names in the lists must match the names of CSV files in the “input” folders, e.g. “ChCl_glycerol” and “glycerol” to “exp_ChCl_glycerol.csv” and “exp_glycerol.csv”.

Different filters can be used to filter the data or different water contents (xw) or molar ratio of the DES-components (rDES), see methods section of the main text for details. These filters and the parameter(s) can be set in "names.py" and are used in the function filtermydataframe(). The lists 'myfilters', 'boundary' and 'variables' are defined for confirming the range of filter and related variables. 'quality' was related with function createmydataframe(), which not only creates a dataframe but also preprocesses data by deleting dataset that number is less than 'quality', e.g. 3.

wrapper.py
The functions used in wrapper.py import functionalities from python scripts in the folder "scripts". Each part of the workflow is summarized into one function (DES mixture ChCl:glycerol and aqueous solution of glycerol will be used as examples):

run_csvtocml() reads data from CSV files like "exp_ChCl_glycerol.csv"and converts them to CML files. Because the CML files that are created by run_csvtocml() are used by other functions later, it must be run in the beginning (Figure 1). The CML files are written to the folder "Results" with name like "ChCl_glycerol.xml" and "glycerol.xml".

run_modellingArrhenius() performs the Arrhenius fits for the data, but only if at least 3 (or another value specified in 'quality') data points are available for each temperature. The viscosity data of each mixture will be read from XML files like "ChCl_glycerol.xml" and "glycerol.xml" (in folder "Results"). The ln(viscosity) will be modelled with Arrhenius equation. Figures of $\ln\eta_0$, $E\eta$ to mole fraction of water are plotted for each mixture and saved in a csv file with name like "Arrhenius_ ChCl_glycerol_filteredby_molar ratio of DES _0.5.csv" and "Arrhenius_glycerol _filteredby_molar ratio of DES _0.0.csv" (in folder "Results"). The plots are written as pngs to the folder "Results" with names like "Arrhenius_fits_ChCl_glycerol.png", "Arrhenius_ChCl_glycerol _filteredby_molar ratio of DES _0.5.png" , "Arrhenius _fits _glycerol.png" and "Arrhenius _glycerol _filteredby_molar ratio of DES_0.0.png". Some of the filenames have been shortened in the publication SI for clarity.

run_modellingVFT() performs the VFT fits for the data, assuming at least 3 or 4 (or another value specified in 'quality') data points are available for each temperature. The viscosity data of each mixture will be read from XML files like "ChCl_glycerol.xml" and "glycerol.xml" (in folder "Results"). The ln(viscosity) will be modelled with VFT model. Figures of parameters A, B, T_0 to mole fraction of water will be plotted for each mixture and saved in a csv file with name like "VFT_ ChCl_glycerol _filteredby_molar ratio of DES _0.5.csv" and "VFT_glycerol _filteredby_molar ratio of DES _0.0.csv" (in folder "Results"). The plots are written as pngs to the folder "Results" with names like "VFT_fits_ChCl_glycerol.png", "VFT_ ChCl_glycerol _filteredby_molar ratio of DES _0.5.png" , "VFT_fits _glycerol.png" and "VFT_glycerol _filteredby_molar ratio of DES_0.0.png". Some of the filenames have been shortened in the publication SI for clarity.

run_overview() will read all the from the Arrhenius fits and the VFT fits data (e.g. "Arrhenius_ ChCl_glycerol_filteredby_molar ratio of DES _0.5.csv" and "VFT_glycerol _filteredby_molar ratio of DES _0.0.csv" in the folder "Results") One overview figure of the ln(viscosity) to mole fraction of water will be plotted for all data for each model (Arrhenius/VFT). The plots are written as "Arrhenius_overview.png" and "VFT_overview.png" to the folder "Results".

run_etaexcess() will fit the excess Eη with the 4th polynomials order for each mixture. The data of each mixture will be read from csv files like "Arrhenius_ ChCl_glycerol_filteredby_molar ratio of DES _0.5.csv" and "VFT_glycerol _filteredby_molar ratio of DES _0.0.csv" (in folder "Results"). Figures of excess Eη to mole fraction of water with a fitted curve will be plotted for each mixture. Besides, the coefficient of determination will also be calculated and shown in figures. The plots are written as pngs to the folder "Results" with name like "Eetaexcess_ChCl_glycerol.png" and "Eetaexcess _glycerol.png".

run_plotviscosities() will plot viscosity data of all mixtures under same temperature to mole fraction of water. The viscosity data of each mixture will be read from XML files like "ChCl_glycerol.xml" and "glycerol.xml" (in folder "Results"). The plots are written as pngs to the folder "Results" with name like "Viscosity_T323.15.png".

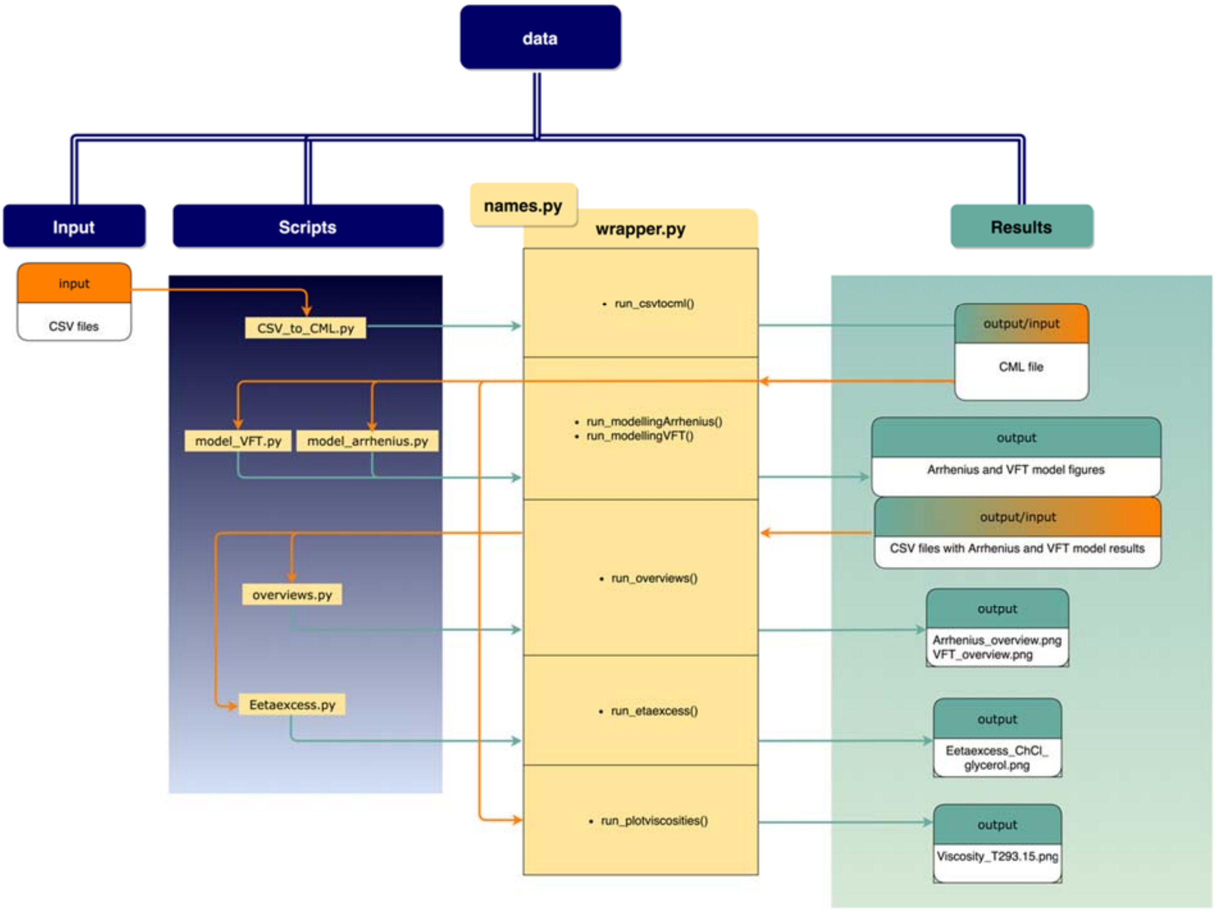

Figure I1: Organization of folders, scripts, and data in them. Folders with dark blue color and their contents must be created before running the workflow: they contain the input files. The main python scripts (yellow) 'names.py' and 'wrapper.py' must be outside the 'scripts' folder. The folder 'Results' (green) will be created by the workflow automatically, so that after execution of 'wrapper.py', there will be 3 folders in 'data' :'Input', 'Scripts' and 'Results'. Input files are colored in orange and output files in green. If a file has both colors, it is output of one task and input of a subsequent task in the workflow.

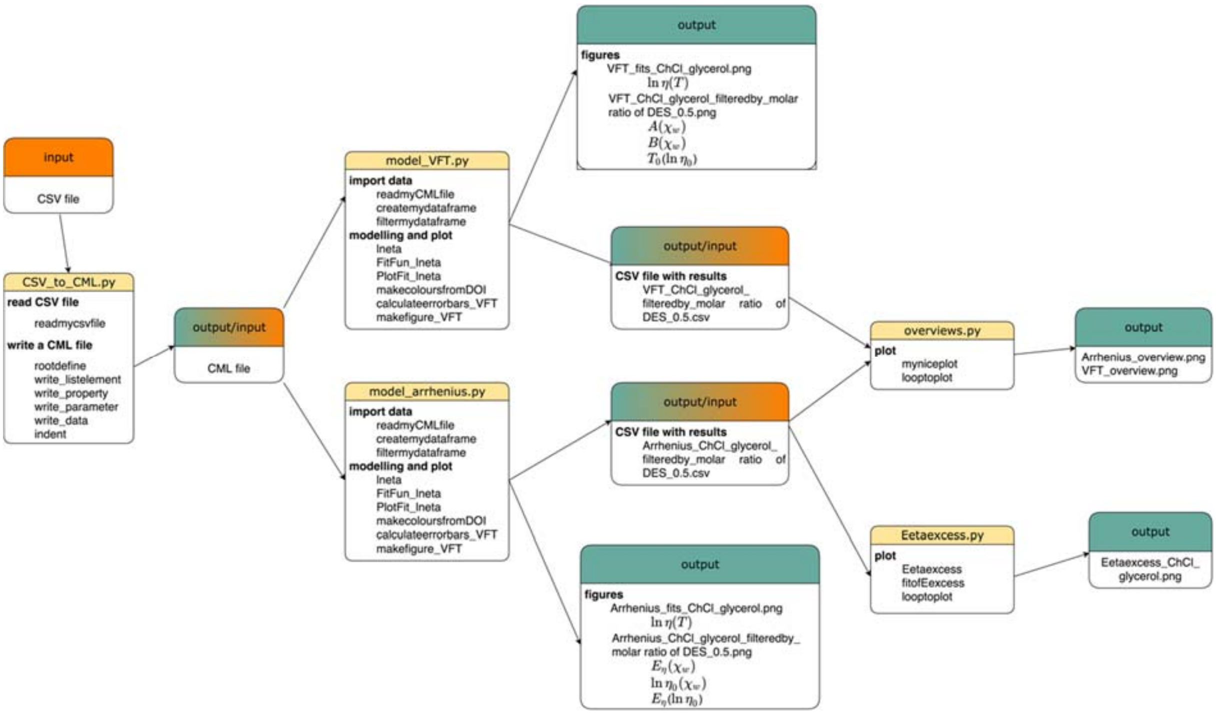

Figure I2: This illustration shows the internal relationships between five python scripts and input(red)/output(green). At the very beginning, CSV files will be read and converted into CML files. The CML files obtained are inputs for scripts "model_VFT.py" and "model_arrhenius.py". "model_VFT.py" and "model_arrhenius.py" own same structure of output, which have two figures and one CSV file with modelled results. The obtained CSV files from modelling will be used to analyze the excess E[illegible] and plot an overview for each model. In boxes of python scripts, typed with normal font are names of function defined by us, while typed with bold font are abstracts. Because this workflow works with 8 mixtures, we use the mixture ChCl:glycerol as an example to represent the name of mixture.
